# Antibodies from convalescent plasma promote SARS-CoV-2 clearance in individuals with and without endogenous antibody response

**DOI:** 10.1172/JCI158190

**Published:** 2022-06-15

**Authors:** Maddalena Marconato, Irene A. Abela, Anthony Hauser, Magdalena Schwarzmüller, Rheliana Katzensteiner, Dominique L. Braun, Selina Epp, Annette Audigé, Jacqueline Weber, Peter Rusert, Eméry Schindler, Chloé Pasin, Emily West, Jürg Böni, Verena Kufner, Michael Huber, Maryam Zaheri, Stefan Schmutz, Beat M. Frey, Roger D. Kouyos, Huldrych F. Günthard, Markus G. Manz, Alexandra Trkola

**Affiliations:** 1 Department of Medical Oncology and Haematology; University Hospital Zurich and University of Zurich; Comprehensive Cancer Center Zurich; Switzerland.; 2 Division of Infectious Diseases and Hospital Epidemiology, University Hospital Zurich, Zurich, Switzerland.; 3 Institute of Medical Virology, University of Zurich, Zurich, Switzerland.; 4 Blood Transfusion Service Zurich, Swiss Red Cross, Zurich, Switzerland.

**Keywords:** COVID-19, Therapeutics, Adaptive immunity, Immunotherapy

## Abstract

**BACKGROUND:**

Neutralizing antibodies are considered a key correlate of protection by current SARS-CoV-2 vaccines. The manner in which human infections respond to therapeutic SARS-CoV-2 antibodies, including convalescent plasma therapy, remains to be fully elucidated.

**METHODS:**

We conducted a proof-of-principle study of convalescent plasma therapy based on a phase I trial in 30 hospitalized COVID-19 patients with a median interval between onset of symptoms and first transfusion of 9 days (IQR, 7–11.8 days). Comprehensive longitudinal monitoring of the virological, serological, and disease status of recipients allowed deciphering of parameters on which plasma therapy efficacy depends.

**RESULTS:**

In this trial, convalescent plasma therapy was safe as evidenced by the absence of transfusion-related adverse events and low mortality (3.3%). Treatment with highly neutralizing plasma was significantly associated with faster virus clearance, as demonstrated by Kaplan-Meier analysis (*P =* 0.034) and confirmed in a parametric survival model including viral load and comorbidity (adjusted hazard ratio, 3.0; 95% CI, 1.1–8.1; *P =* 0.026). The onset of endogenous neutralization affected viral clearance, but even after adjustment for their pretransfusion endogenous neutralization status, recipients benefitted from plasma therapy with high neutralizing antibodies (hazard ratio, 3.5; 95% CI, 1.1–11; *P =* 0.034).

**CONCLUSION:**

Our data demonstrate a clear impact of exogenous antibody therapy on the rapid clearance of viremia before and after onset of the endogenous neutralizing response, and point beyond antibody-based interventions to critical laboratory parameters for improved evaluation of current and future SARS-CoV-2 therapies.

**TRIAL REGISTRATION:**

ClinicalTrials.gov NCT04869072.

**FUNDING:**

This study was funded via an Innovation Pool project by the University Hospital Zurich; the Swiss Red Cross Glückskette Corona Funding; Pandemiefonds of the UZH Foundation; and the Clinical Research Priority Program “Comprehensive Genomic Pathogen Detection” of the University of Zurich.

## Graphical abstract

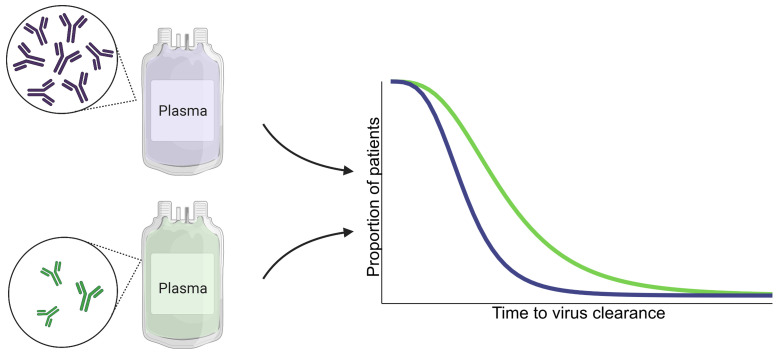

## Introduction

Neutralizing antibodies are recognized as a principal correlate for protection induced by SARS-CoV-2 vaccines (1, 2) and have been used for antiviral treatment as the active component in convalescent plasma therapy (3–9) and as monoclonal antibody (mAb) therapeutics (10, 11). Unless applied very early, in most clinical trials antibody-based SARS-CoV-2 therapies have not achieved the substantial disease-modulating effect initially hoped for. The influence of most plasma- and neutralizing mAb–based interventions was limited to early infection at best (5, 12, 13). Accordingly, with one exception (14), therapeutic mAbs are thus far predominantly applied in nonhospitalized, early infection for people at risk (15–17).

Trials of convalescent plasma therapy were initiated early in the pandemic, and rapidly bolstered by an FDA emergency use authorization (18–22). However, despite numerous clinical trials, the utility of convalescent plasma in COVID-19 remained uncertain, with some studies and meta-analyses reporting no effects or only modest effects (19–21, 23–29) and some indicating benefit (3–9, 30–33). Differences in study design, however, limit direct comparative outcome analyses across studies. Analysis of the effective plasma antibody titers applied, the timing of administration, and the recipient’s immune status will be critical to resolve inconsistencies.

The presumed active component of SARS-CoV-2 convalescent plasma, neutralizing antibodies, is commonly not high-titered (34, 35) and substantially diluted upon transfusion. Considering the immediate consumption of these antibodies in virus-immune complexes and their natural half-life, the activity of transfused antibodies is likely limited to a comparatively short period of time.

Therefore, in addition to mortality, other outcome and analytical parameters must be determined to establish the basic efficacy of plasma antibody therapy. Since comprehensive assessment of clinical and laboratory parameters is not feasible in large-scale trials, small investigational proof-of-principle studies are needed to define suitable outcome and parameter measures. With this goal in mind, we used the framework of a phase I study to conduct a proof-of-principle study (ClinicalTrials.gov NCT04869072) to evaluate the safety of convalescent plasma therapy and explore donor plasma antibody and outcome parameters for future efficacy studies. In contrast to previous studies, the study population comprised 34% immunocompromised patients, a clearly underrepresented population in COVID clinical trials. Next to the conventional outcome and clinical parameters, we focused on a comprehensive assessment of the recipient seroconversion status and the SARS-CoV-2 antibody profile of donor plasma to elucidate which parameters are associated with viral clearance.

## Results

### Study design.

**Figure 1:**
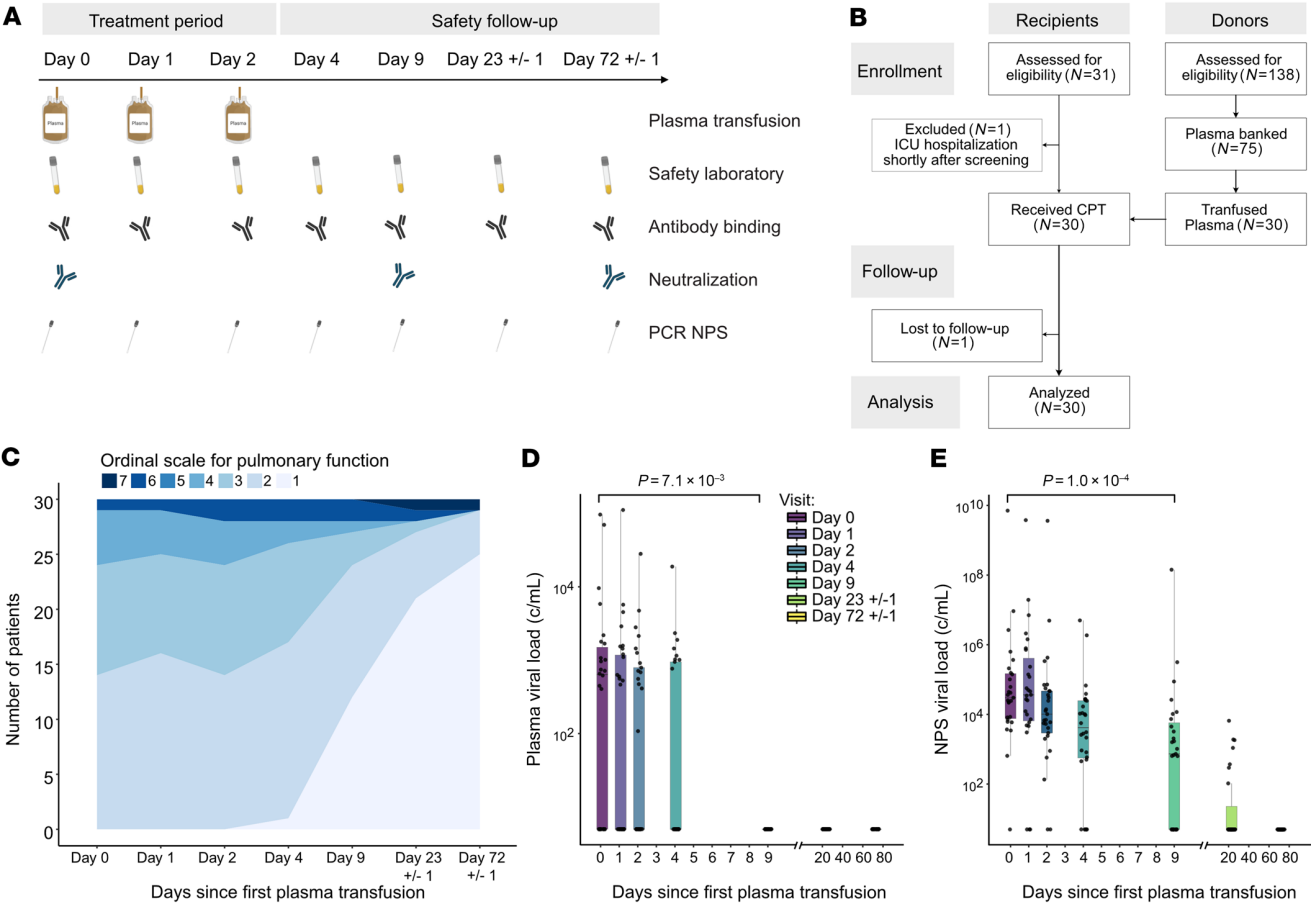
Study design and clinical and virological assessment. (**A**) Schematic depiction of the study design, including timeline of consecutive treatment with convalescent plasma units and clinical and laboratory assessments. PCR NPS, PCR from nasopharyngeal swab. Figure created with BioRender (biorender.com). (**B**) Study flow chart. CPT, convalescent plasma therapy. (**C**) Longitudinal clinical assessment of trial participants (*n =* 30), with a 7-category ordinal scale for pulmonary function. 1: Usual activities with minimal/no symptoms. 2: No supplemental oxygen; symptomatic and unable to undertake usual activities. 3: Supplemental oxygen <4 L/min. 4: Supplemental oxygen ≥4 L/min. 5: Noninvasive ventilation or high-flow oxygen. 6: Invasive ventilation, extracorporeal membrane oxygenation, mechanical circulatory support. 7: Death. (**D** and **E**) Assessment of viral load. Longitudinal viral RNA concentrations (copies/mL) in plasma (**D**) and NPS (**E**) in trial participants (*n =* 30).

To investigate the potential of convalescent plasma therapy, we conducted a nonrandomized, open-label, phase I clinical trial, “Convalescent Plasma Therapy – Zurich Protocol (CPT-ZHP)” (NCT04869072), that included a comprehensive SARS-CoV-2 antibody profiling of donor plasma alongside a longitudinal monitoring of laboratory and clinical parameters (Figure 1A and Supplemental Tables 1–3; supplemental material available online with this article; https://doi.org/10.1172/JCI158190DS1). At the time of study initiation in April 2020, the study was ranked as a first-in-human study by Swiss authorities, necessitating a focus on safety and excluding the formation of a no-treatment group. Next to safety as a primary outcome, this proof-of-principle study was tailored to allow a within-study efficacy assessment. We conducted an extensive monitoring of parameters, to determine what effect transfused SARS-CoV-2 plasma antibodies have on the virological and disease status (secondary outcomes) (Supplemental Table 4). Patients received 3 units of plasma (200 mL each) of a single donor on 3 consecutive days (in total, 600 mL) followed by extensive clinical and laboratory marker monitoring over 70 days (Figure 1A). Consecutive administrations of smaller plasma volumes (200 mL each) were chosen to limit the risk of transfusion-related adverse events while allowing high dosing of convalescent plasma. The study protocol did not specify a threshold for SARS-CoV-2 serum antibodies in donor plasma for several reasons. At the time of study initiation, April 2020, the role of protective, neutralizing antibodies had not been ascertained, and validated serology and neutralization tests were not yet available. Setting arbitrary thresholds for SARS-CoV-2 reactivity without knowing relevant protective levels was thus considered as problematic, since, if wrongly set, this may limit the potential to retrieve information on the therapeutic effect of SARS-CoV-2 antibodies. The study design thus allowed for inclusion of plasma donors without prior screening for SARS-CoV-2 antibody levels. This ascertained that plasma used for therapy would capture a range of SARS-CoV-2 levels enabling a post hoc analysis of the influence of antibody dose on outcome.

### Study population and baseline characteristics.

Thirty SARS-CoV-2–infected patients, hospitalized with COVID-19, were enrolled between April and December 2020 at a single trial center, the University Hospital Zurich, Zurich, Switzerland, according to the approved study inclusion and exclusion criteria (Figure 1B and Supplemental Table 2). All trial participants showed radiological signs of COVID-19 pneumonia at inclusion, with 18/30 (60%) requiring oxygen supplementation but not treatment in an intensive care unit. Baseline characteristics at inclusion (Table 1 and Supplemental Table 5) were typical for COVID-19 and reflected the demographic distribution observed for hospitalized patients in Switzerland in 2020 (36, 37). The median age at inclusion was 63.5 years (IQR, 58.2–68.5), 10/30 participants (33%) were women, and 22/30 participants (73%) presented with 1 or more comorbidities (Table 1 and Supplemental Table 5). Thirty-three percent (10/30) of patients had immunosuppression, including immunodeficiency (17%), cancer (10%), and solid organ transplantation. During the trial period, mostly SARS-CoV-2 lineages derived from B.1, harboring D614G but otherwise closely related to the original Wuhan-Hu-1 strain (MN908947.3), were prevalent in Switzerland (GISAID; www.gisaid). Full genome sequencing of SARS-CoV-2 in nasopharyngeal swabs (NPSs) of 26 plasma recipients confirmed this (Supplemental Figure 1 and Supplemental Table 5).

In addition to plasma therapy, all patients received the standard COVID-19 treatment recommended in Switzerland at the respective time of admission. This was initially limited to symptomatic control, supportive care, and oxygen therapy and was later extended to include therapy with remdesivir (12, 38, 39) and/or steroids (40) when these options became available in Switzerland (Table 1 and Supplemental Table 5). Five patients (17%) received systemic steroid therapy, four (13%) were treated with remdesivir, and one (3.3%) received combination therapy with systemic steroids and remdesivir (Table 1 and Supplemental Table 5).

### Convalescent plasma therapy and safety assessment.

Participants received a total of 600 mL, split as 200 mL plasma units of the same ABO-compatible plasma donor on 3 consecutive days. In line with numerous other studies that attest to the safety of convalescent plasma therapy in COVID-19 (41–43), we observed no transfusion-related adverse events (Supplemental Tables 6 and 7). Adverse events that were ranked as related to COVID-19 or other underlying diseases but not to plasma therapy were observed in 6/30 individuals (20%), in the range expected for the included COVID-19 stage (Supplemental Table 7 and ref. 22).

One patient (patient 15; Supplemental Table 5) with chronic lymphocytic leukemia died from bacterial, hospital-acquired pneumonia by day 12. No other deaths occurred by study completion, resulting in an overall mortality rate of 3.3% (Supplemental Table 7) within 72 days after study enrollment. Median duration of hospitalization was 8 days (IQR, 6–13). To include an outcome measure that allows a gradual assessment of disease progression and cure, we longitudinally assessed the patients’ health status by a 7-category ordinal scale for pulmonary function as previously described (refs. 44, 45, and Supplemental Table 8). The function score improved gradually, with 25/30 (83%) patients reaching full pulmonary function by study completion (Figure 1C and Supplemental Table 9). Only 2 participants required intensive care over the trial period and needed mechanical ventilation (Figure 1C and Supplemental Table 9). Notably, we observed overall a rapid improvement in respiratory rate, oxygen saturation, and body temperature at day 9, i.e., 1 week after the last plasma dose (Supplemental Figure 2A and Supplemental Table 10). Laboratory markers of inflammation progressively improved in the majority of participants to reach reference values at study completion (day 72) (Supplemental Figure 2B and Supplemental Table 10). C-reactive protein levels in particular showed a rapid decline following plasma therapy (Supplemental Figure 2B and Supplemental Table 10). Coagulopathy, indicated by increased D-dimer and fibrinogen levels, has been frequently observed in patients with COVID-19 and is associated with subsequent thromboembolic events and severe outcomes (46–50). Notably, fibrinogen was elevated at baseline in all patients but was already significantly decreased by day 4 (*P =* 0.0063, 2-sided, paired *t* test), whereas D-dimer levels were elevated only in a fraction of participants (21/28, 75%) and remained at comparable levels throughout.

To monitor virological improvement, we measured SARS-CoV-2 viral load in blood and NPSs (Figure 1, D and E). Median log_10_ baseline viral load in NPSs was 4.5 (IQR, 3.9–5.2), with 16/30 individuals presenting with measurable SARS-CoV-2 viremia in plasma. Viral load in both specimens rapidly decreased in line with the normalization of clinical parameters (Figure 1, D and E).

### Antibody profiling of SARS-CoV-2 convalescent plasma.

**Figure 2:**
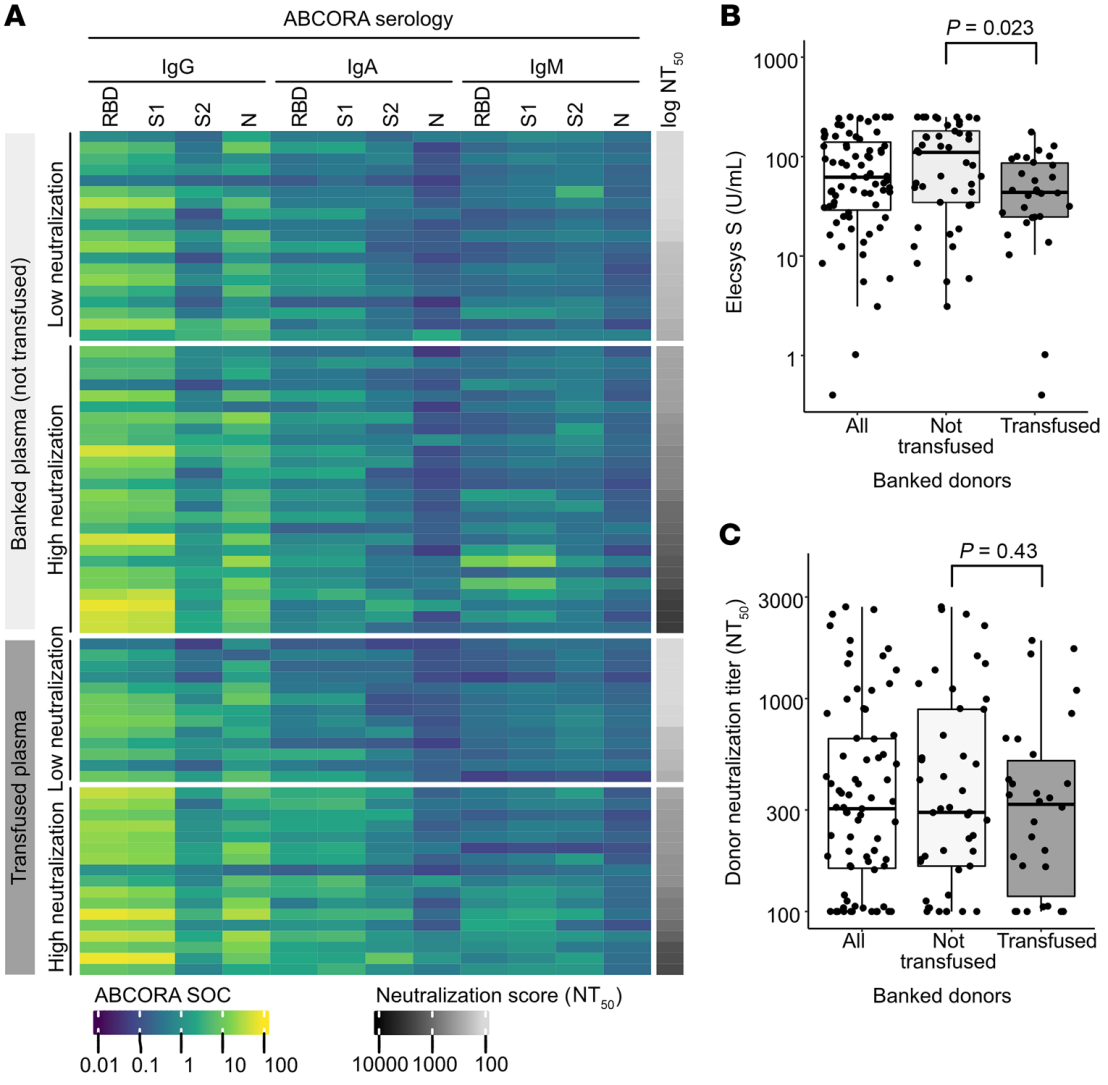
Antibody characteristics of convalescent donor plasma. (**A**) Profiling of donor plasma for neutralization activity and seroreactivity to SARS-CoV-2 antigens. Data of all banked donors (transfused, *n =* 30; not transfused, *n =* 45) are shown. Binding antibody reactivity was measured in the ABCORA test (readout median fluorescence intensity signal over cutoff [SOC]). Fifty percent neutralization titers (NT_50_) were measured against Wuhan-Hu-1 pseudotyped virus. Donor plasmas are stratified into plasmas with high (NT_50_ > 250) and low (NT_50_ ≤ 250) neutralization potency. (**B**) Antibody binding titers in banked donor plasma measured by the Elecsys S assay (U/mL). (**C**) NT_50_ titers against Wuhan-Hu-1 pseudotyped virus in banked plasma donors. (**B** and **C**) Two-sided, unpaired *t* test comparing the transfused and non-transfused plasma.

During the study, a total of 75 plasma donations were collected from convalescent male donors to ascertain the availability of ABO-compatible plasma (Table 2 and Supplemental Table 10). Post hoc analysis of the SARS-CoV-2 serological responses in this plasma cohort with the sensitive multiplex seroprofiling test ABCORA showed a heterogeneous pattern prototypic for SARS-CoV-2 infection ranging from high responses with IgG, IgA, and IgM reactivity to spike proteins (S1, RBD, and S2) and nucleoprotein (N) to low reactivity (Figure 2A). This pattern of high and low serum responses was confirmed by monitoring of total Ig against the receptor-binding domain (RBD) in the Elecsys S assay (Roche Diagnostics GmbH) (Figure 2B). Neutralizing titers against Wuhan-Hu-1 pseudovirus in donor plasma ranged from no neutralization activity to a titer of 2700 (Figure 2C).

### Impact of convalescent plasma antibody on viral clearance.

Release from the hospital in our trial depended not only on the health status but also on de-isolation rules that were adapted over time by authorities. Time to hospital discharge could therefore not be used as an endpoint. We therefore directly assessed the impact on virological improvement and investigated whether and which SARS-CoV-2–specific donor plasma antibody parameters are associated with viral clearance. Neutralizing antibodies are the presumed active antiviral component of convalescent plasma. Effective treatment should therefore result in decreasing viral load.

**Figure 3:**
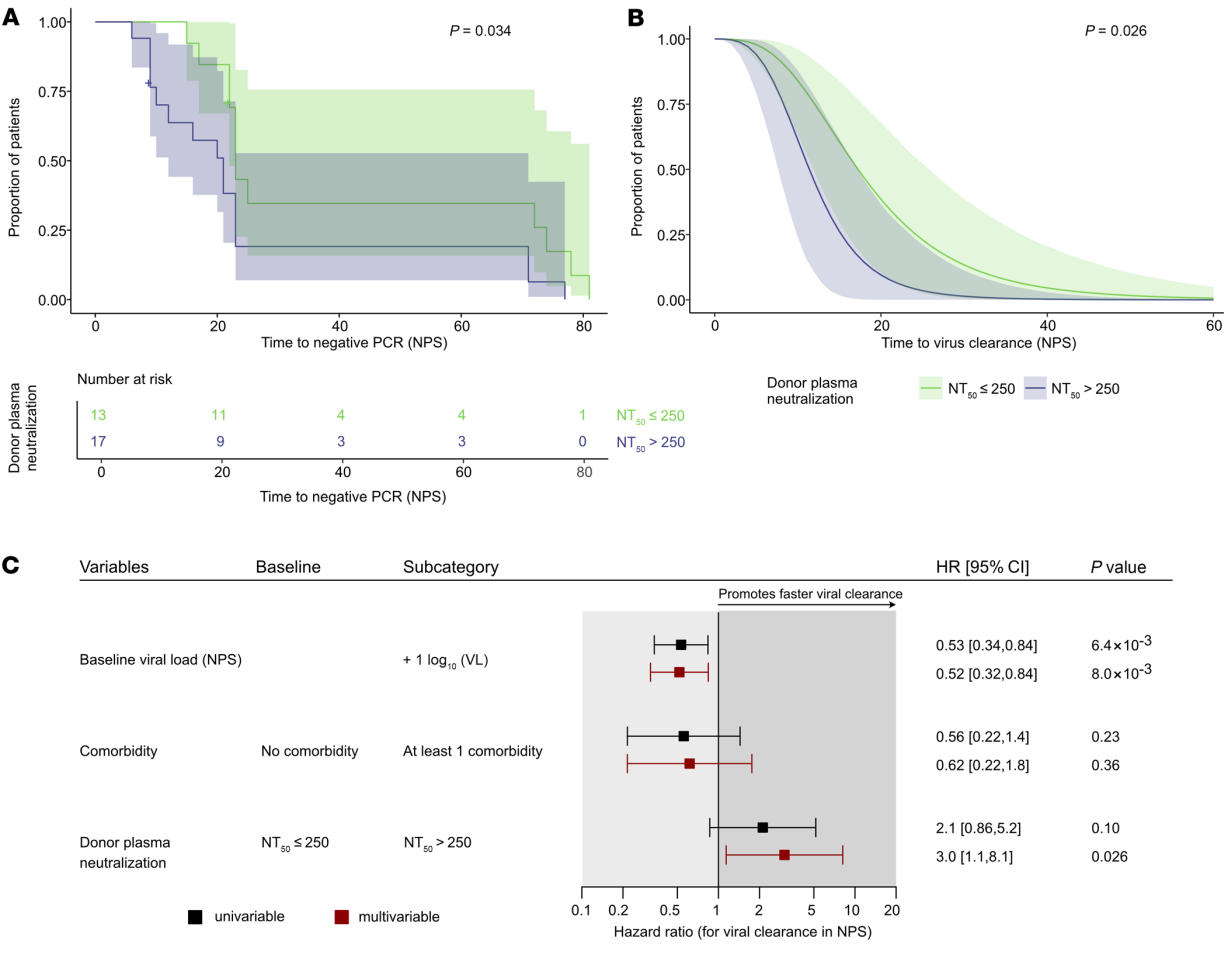
Treatment with highly neutralizing plasma leads to faster viral clearance. (**A** and **B**) Assessment of the time (days) to viral clearance in NPSs in plasma recipients (*n =* 30) according to the level of neutralization potency of the received convalescent donor plasma. High neutralization activity is set as NT_50_ > 250, low neutralization activity as NT_50_ ≤ 250. (**A**) Kaplan-Meier curves compared by log-rank test. (**B**) Survival function estimate with a parametric model for interval-censored data. The parametric estimate is adjusted for the baseline NPS viral load and the presence of any comorbidity. Depicted survival curves of recipients of high- and low-neutralizing donor plasma correspond to the predicted viral clearance in individuals without comorbidity and with a baseline viral load (NPS) equal to the median viral load observed among the 30 patients. (**C**) Forest plot corresponding to **B**, showing the hazard ratios of the univariable (black) and the multivariable (red) model of time to viral clearance in NPSs for convalescent donor plasma neutralization level (low/high), baseline viral load, and the presence of comorbidity.

In conformity with analyses conducted by the Expanded Access Program and the FDA (51, 52), we classified high and low neutralizing plasma by a 50% neutralization titer (NT_50_) of 250 and stratified patients accordingly. Baseline characteristics of trial participants receiving high- and low-titer plasma were found to be very comparable (Table 1). Treatment with highly neutralizing plasma was associated with faster viral clearance in NPSs, as shown by a basic Kaplan-Meier analysis (*P =* 0.034; Figure 3A), but not with time to hospital discharge (Supplemental Figure 3). We next verified this result in a parametric model that allowed for interval-censored data for the SARS-CoV-2 antibody measurement and adjusted for 2 recipient baseline parameters. We adjusted for viral load in NPSs because higher viral loads are likely to require longer to clear. We also considered that viral clearance was mediated by both the patient’s endogenous immune response and the supplied convalescent plasma. We further adjusted for comorbidities, as several trial participants had underlying diseases that can lead to impaired immune function (Supplemental Table 5). The parametric model including NPS viral load and comorbidity confirmed the effect of convalescent plasma content on viral clearance in NPSs (adjusted hazard ratio, 3.0; 95% CI, 1.1–8.1; *P =* 0.026; Figure 3, B and C). We verified these observations in a series of sensitivity analyses. Exclusion of 3 individuals who were incapable of mounting an antibody response to SARS-CoV-2 did not alter the result (adjusted hazard ratio, 2.8; 95% CI, 1.0–7.7; *P =* 0.046; Supplemental Figure 4, A and B). Excluding remdesivir-treated individuals (*n =* 4) from the hazard ratio analysis, we observed an even higher impact of plasma neutralizing activity on viral clearance (adjusted hazard ratio, 4.8; 95% CI, 1.6–14; *P =* 0.0056; Supplemental Figure 4, C and D). The outcome further remained unchanged with (a) exclusion of both individuals without endogenous SARS-CoV-2 antibodies and individuals with remdesivir treatment (*n =* 6) (Supplemental Figure 4, E and F), (b) exclusion of systemic corticosteroid–treated patients (*n =* 5) (Supplemental Figure 4, G and H), or (c) exclusion of individuals based on all 3 criteria (systemic corticosteroids, remdesivir, and incapability of mounting antibody response; *n =* 10) (Supplemental Figure 4, I and J). On the basis of these analyses, we concluded that high neutralizing activity in convalescent plasma promotes rapid viral clearance.

**Figure 4:**
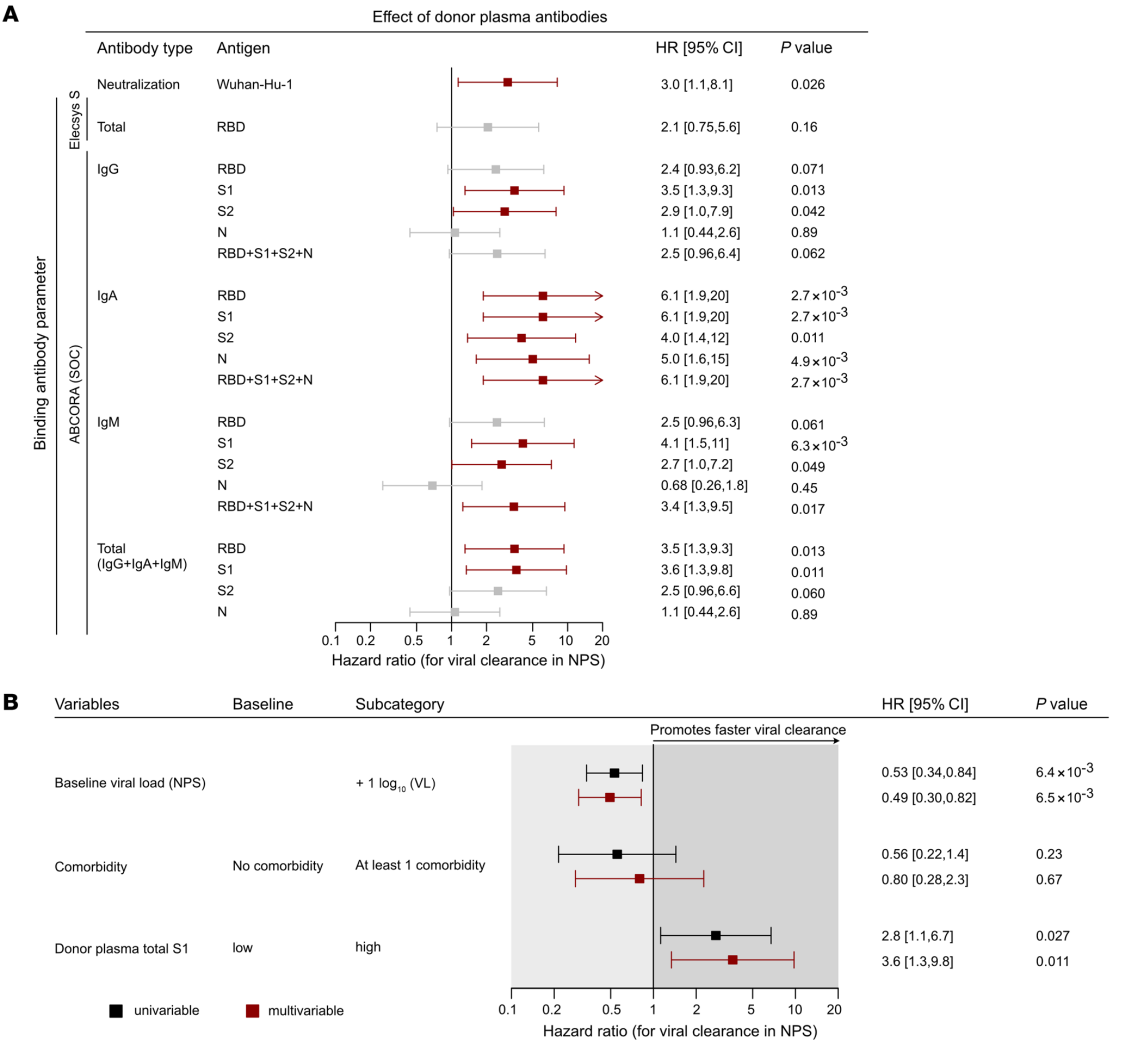
Spike-specific binding and neutralizing antibodies in convalescent donor plasma are linked with rapid viral clearance. (**A**) Impact of convalescent donor plasma antibody parameters on the time to viral clearance in recipients (*n =* 30) was assessed by multivariable parametric survival models. Hazard ratios for individual antibody reactivities adjusted for the presence of comorbidity and the baseline viral load (NPS) are shown. Significant results are marked in red. Low and high binding activity for each individual binding antibody parameter measured in the Elecsys S test (total RBD) and for the ABCORA test parameters is stratified by the respective median binding reactivity. Low and high neutralization activity of transfused convalescent donor plasma is stratified by an NT_50_ of 250. (**B**) Forest plot depicting hazard ratios of univariable (black) and multivariable (red) models of time to viral clearance including total S1 (sum of ABCORA IgG, IgA, and IgM reactivity with S1) stratified by the median binding reactivity. Multivariable analyses are corrected for baseline viral load (NPS) and the presence of comorbidity.

Considering that most convalescent plasma studies did not assess neutralizing activity in convalescent plasma but relied on more readily available serological assays, we next examined whether any of the antibody types among the 12 SARS-CoV-2 reactivities detected by ABCORA seroprofiling or the Elecsys S assay readout confirmed the finding for neutralizing antibodies. To this end, we performed a series of survival analyses assessing the effect of each individual antibody reactivity, as well as composite total antibody reactivities, on time to viral clearance (Figure 4A, Supplemental Figure 5, and Supplemental Table 11). In each of these analyses, we stratified plasma according to the median reactivity into a high- and a low-reactivity treatment group and again controlled for remdesivir treatment (Supplemental Figure 6) and immune suppression (Supplemental Figures 7 and 8) in sensitivity analyses. Most neutralizing SARS-CoV-2 antibodies target the RBD and the receptor-binding motif within the RBD, leaving only a comparatively small fraction of neutralization to S1 trimer-specific, spike N-terminal domain, and spike S2 antibodies (53–59). Accordingly, we initially focused on RBD responses but did not detect a differential effect of plasma on viral clearance when stratifying based on the Elecsys S assay (Figure 4A and Supplemental Figure 9). This was in stark contrast to reactivities determined by the ABCORA seroprofiling test, by which high S1 IgG, IgA, and IgM levels and high RBD IgA levels were associated with faster viral clearance irrespective of whether remdesivir-treated individuals were included (Figure 4A and Supplemental Figure 6). Particularly notable were IgA responses that displayed the highest impact on rapid viral clearance across all antigens. Owing to their sequential evolution upon seroconversion, reactivities within an antibody class are expected to be correlated to a certain degree. This was also evident in our plasma donor cohort (Supplemental Figure 10). Based on the observed high association of S1 reactivities with viral clearance, we selected the composite S1 value comprising IgG, IgA, and IgM S1 activity (total S1) as a parameter to be included in further analysis (Figure 4B).

**Figure 5:**
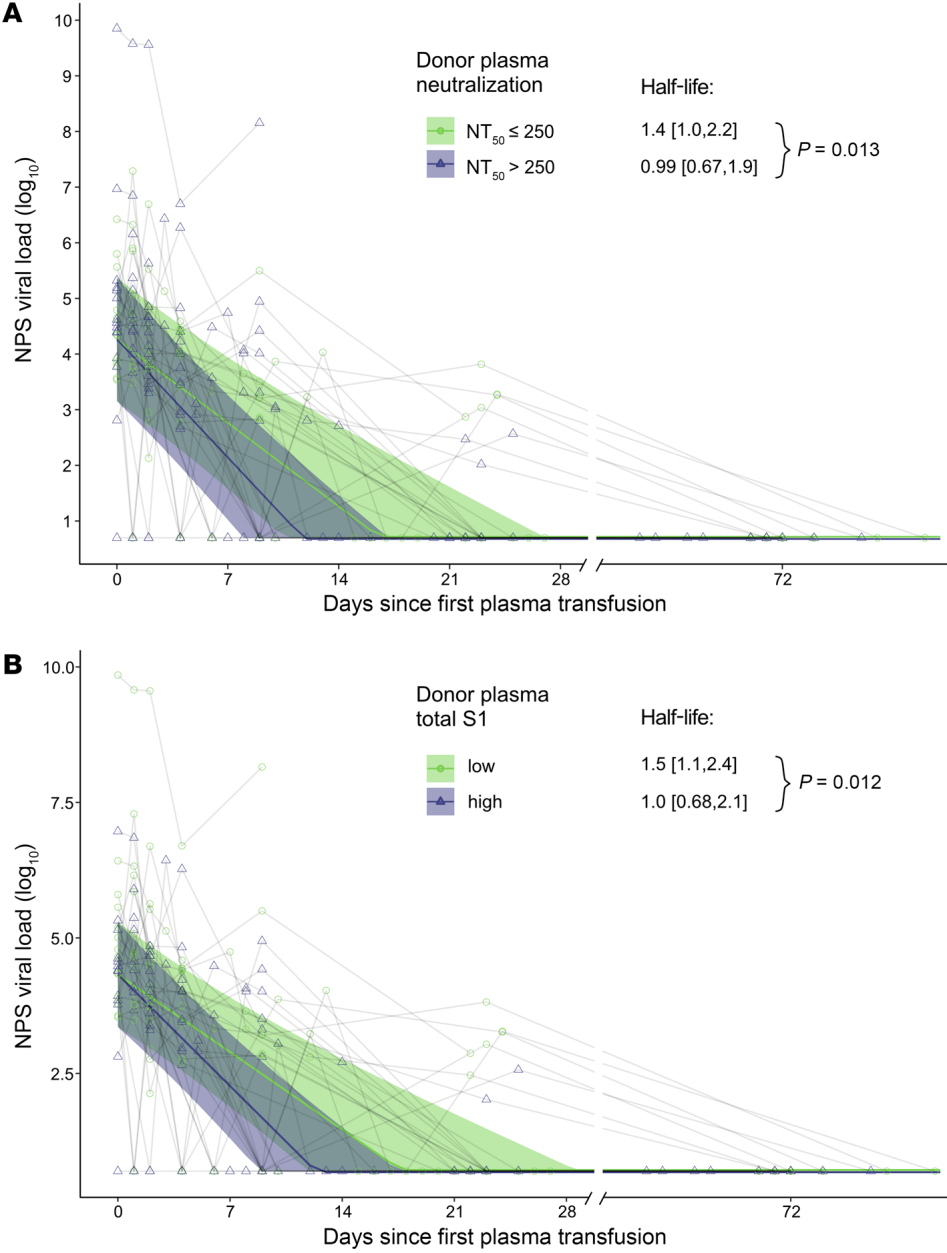
High-neutralizing plasma leads to faster virus decay in NPSs. (**A** and **B**) Censored regression model estimating decay rate of viral load (log_10_ viral load) in NPSs in recipients (*n =* 30) from time of treatment initiation according to the received convalescent donor plasma with respect to neutralizing antibody content (low neutralization, NT_50_ ≤ 250, light green; high neutralization, NT_50_ > 250, purple) (**A**) or level of binding antibodies as defined by the ABCORA test total S1 values (sum of IgG, IgA, and IgM reactivity with S1) (**B**). Low and high total S1 binding is stratified by the median binding reactivity. Significance was assessed using a 2-sided *t* test.

We further quantified the effect of plasma therapy on virus decay dynamics using censored regression models. We found that half-lives of virus load in NPSs in recipients of high neutralizing plasma were shorter, confirming the results from the hazard analysis (half-lives: 1.4 days [95% CI, 1.0–2.2] when NT_50_ ≤ 250 vs. 0.99 days [95% CI, 0.67–1.9] when NT_50_ > 250, *P =* 0.013) (Figure 5A). This effect was also evident when remdesivir-treated individuals were excluded, and with stratification for S1 reactivity (total S1) (Figure 5B and Supplemental Figure 11, A and B).

### Endogenous neutralizing antibodies control plasma viremia.

**Figure 6:**
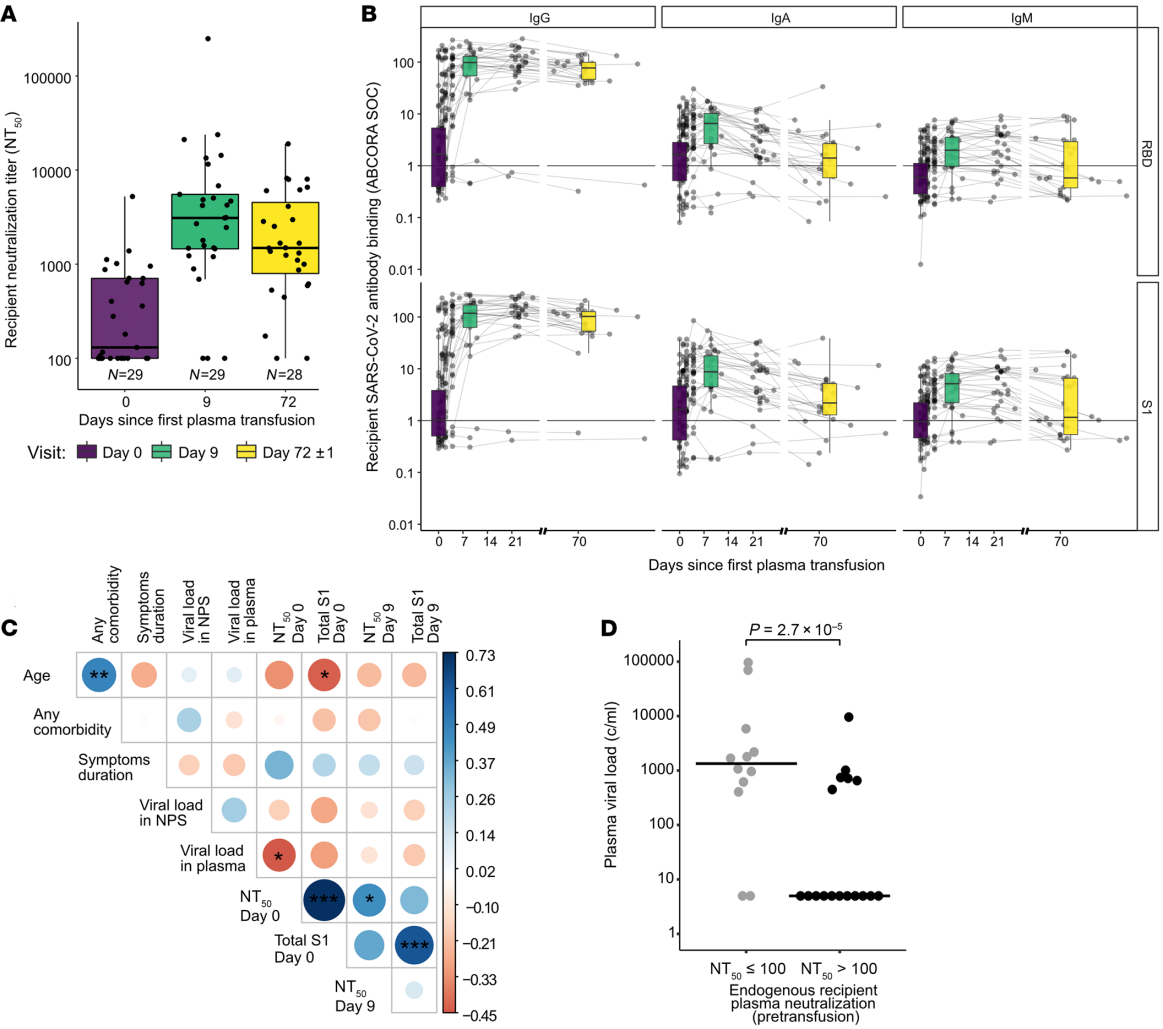
Recipients’ endogenous neutralizing antibodies efficiently control plasma viremia. (**A**) Recipients’ pretransfusion endogenous 50% plasma neutralization titers (NT_50_) against Wuhan-Hu-1 pseudovirus. For each time point (days 0, 9, 72) the number of patients with available sample is indicated. Box plots depict the interquartile ranges, with vertical lines (whiskers) representing a distance of 1.5 times the interquartile range below the first quartile and above the third quartile. (**B**) Recipients’ longitudinal binding antibody activity at baseline (day 0), day 9, and day 72 assessed with the multiplex SARS-CoV-2 ABCORA 2 test. Sample numbers per time point are as shown in **A**. Signal over cutoff (SOC) values of IgG, IgA, and IgM against RBD and S1 are shown. Box plots indicate the interquartile ranges,with vertical lines (whiskers) representing a distance of 1.5 times the interquartile range below the first quartile and above the third quartile. (**C**) Spearman’s correlation matrix assessing correlation between age; comorbidities; viral load in NPS (copies/mL) and blood (binary yes/no); and neutralization titer (NT_50_) and total S1 (SOC values) at day 0 and day 9. Levels of significance were assessed by asymptotic *t* approximation of Spearman’s rank correlation. Color shading denotes correlation coefficient. Levels of significance: *P < 0.05, **P < 0.01, ***P < 0.001. (**D**) Group comparison of plasma viral load from recipients stratified by presence of pretransfusion endogenous neutralization activity (baseline *d* = 0) (no neutralization, NT_50_ ≤ 100; neutralization activity, NT_50_ > 100). Levels of significance were calculated by Wilcoxon’s rank sum test.

Our trial focused on individuals with COVID-19 with severe pulmonary manifestation requiring hospitalization but not intensive care. The median interval between the onset of symptoms and the first transfusion was 9 days (IQR, 7–11.8 days; Table 1). To rate the seroconversion status of trial participants, we monitored the evolution of SARS-CoV-2 antibodies at baseline and selected time points throughout the trial (Figure 6, A and B, and Supplemental Figure 12). Seroprofiling with the ABCORA test indicated a wide range of seroconversion among participants at baseline ranging from low-level partial responses to full-blown seroconversion with high IgG anti-spike levels. Overall, trial participants were in a relatively early stage of the immune response as illustrated by increasing IgM, IgA, and IgG levels (Figure 6B). Twelve individuals had no detectable neutralization activity at baseline (Figure 6A), but with the exception of the 3 individuals who completely lacked the capacity to mount an antibody response (Supplemental Figure 13), neutralizing and binding antibodies increased in the majority of participants from baseline to day 9 and plateaued thereafter (Figure 6, A and B, and Supplemental Figure 12). Presence of neutralizing antibodies had an impact on the virological status of the recipient. Detectable SARS-CoV-2 in blood was inversely linked with neutralization activity at baseline (Spearman’s correlation, *P =* 0.015; Figure 6C). Stratifying patients according to the presence of pretransfusion endogenous neutralization activity (*n =* 29 patients with available data; Supplemental Table 12) corroborated the effect of the endogenous neutralizing response on suppression of systemic viremia in the early phase of the infection in an alternate analysis (Wilcoxon’s rank sum test, *P =* 0.000027; Figure 6D). We therefore concluded that the endogenous pretransfusion serological status of the recipient, particularly neutralizing responses, and the virological status must be considered in evaluating SARS-CoV-2 antiviral therapies.

### Impact of the endogenous serological status on the outcome of plasma therapy.

**Figure 7:**
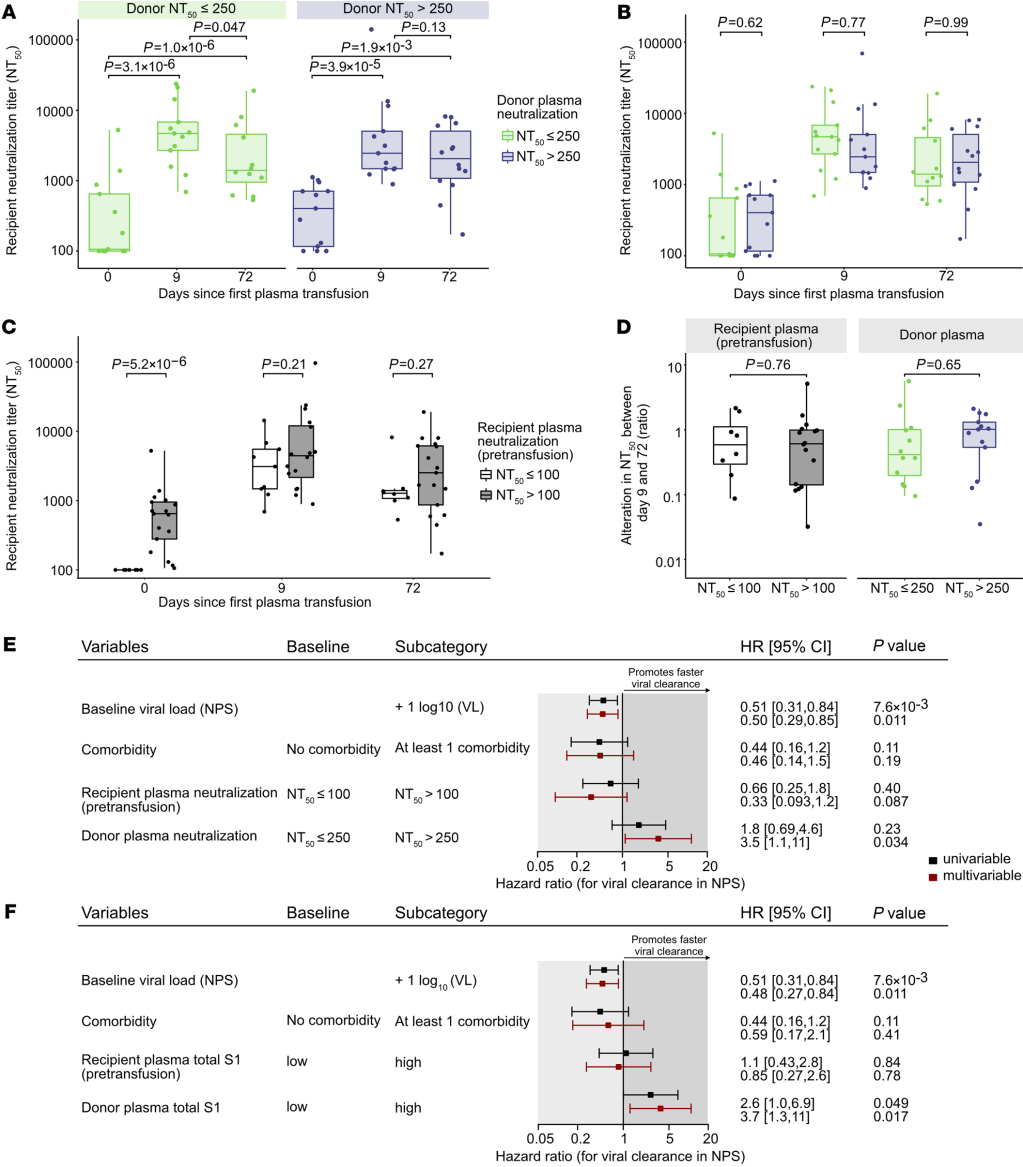
Influence of recipients’ endogenous and convalescent plasma antibodies on viral clearance. (**A**) Analysis of recipients’ plasma neutralization activity before and after transfusion of convalescent plasma. NT_50_ titers against Wuhan-Hu-1 pseudotyped virus at baseline (day 0, *n =* 26), day 9 (*n =* 26), and day 72 (*n =* 26) are depicted. Recipients are stratified by neutralizing levels of transfused convalescent donor plasma (left: low NT_50_ donor plasma ≤ 250; right: high NT_50_ > 250). Levels of significance were calculated by 2-sided, paired *t* test. (**B**) Longitudinal comparison of NT_50_ activity in low/high donor plasma NT_50_ groups at days 0, 9, and 72. Levels of significance were calculated by 2-sided, unpaired *t* test. (**C**) Longitudinal comparison of the evolution of recipient neutralization activity (no neutralization, NT_50_ ≤ 100; neutralization activity, NT_50_ > 100). Levels of significance were calculated by unpaired *t* test. (**D**) Alteration in NT_50_ between days 9 and 72 (ratio) according to the donor and recipient baseline neutralization. Levels of significance were calculated by 2-sided, unpaired *t* test. (**E** and **F**) Forest plot showing hazard ratios of univariable and multivariable survival models of time to viral clearance (*n =* 26). Both **E** and **F** test for baseline viral load and comorbidity; **E** tests additionally for neutralization level in donor plasma and recipients’ pretransfusion plasma, and **F** tests additionally for S1 antibody level in donor plasma and in recipients’ pretransfusion plasma.

Viral clearance in the context of the study needs to be viewed as the composite of the patients’ immunity and the activity of therapeutic plasma. We therefore investigated whether endogenous and exogenous neutralizing antibody activity coinfluence each other. Of the 29 individuals with available pretransfusion neutralization data, 3 immunocompromised individuals incapable of mounting antibody responses were excluded (Supplemental Figure 13), resulting in a subcohort of 26 individuals for these analyses. Neutralization titers significantly increased in both recipients of high- and low-neutralization plasma by day 9 (Figure 7A) to comparable levels (Figure 7B), suggesting that by this time point the measured activity is dominated by the endogenous neutralization response and transfused antibodies have a marginal contribution. Neutralization activity increased in immunocompetent individuals with and without endogenous neutralization activity at baseline and reached comparable levels by day 9 (Figure 7C). To probe whether the endogenous neutralization activity before transfusion or the neutralization antibody content of the donor plasma has an influence on the durability of the neutralization response, we assessed the relative change in neutralization titers between days 9 and 72. Groups stratified for low and high recipient and donor plasma neutralization activity showed similar patterns of increasing and decreasing neutralization activity (Figure 7D). No differences in the durability of neutralization activity were detected by day 72 in either subgroup, supporting the notion that plasma treatment has a transient impact and no immunomodulatory effect.

Finally, we tested for the combined effect of neutralization activity in the donor and recipient plasma on viral clearance. We hypothesized that viral clearance will be already ongoing in individuals who have neutralizing antibodies at baseline. Strong effects on virus clearance during plasma therapy may therefore be observed in individuals with no baseline neutralization activity, for whom the onset of endogenous neutralizing antibody and the convalescent plasma treatment overlap. Therefore, to distinguish the effect of donor plasma on viral clearance, we thought it prudent to consider the effect of recipient neutralization activity. To address this, we adjusted the parametric survival model shown in Figure 3C by accounting for recipient endogenous neutralization activity pretransfusion in addition to baseline viral load, comorbidities, and donor plasma neutralization activity.

High neutralization activity in convalescent plasma was associated with faster clearance both when the immunocompromised individuals were excluded (*n =* 26; hazard ratio, 3.5; 95% CI, 1.1–11; *P =* 0.034; Figure 7E) and in the full cohort (*n =* 29; hazard ratio, 4; 95% CI, 1.3–13; *P =* 0.017; Supplemental Figure 14A). This demonstrates a positive impact of plasma therapy even in individuals who already have mounted an antibody response to SARS-CoV-2. Individuals who lacked neutralization activity at baseline showed a trend toward more rapid clearance, suggesting a strong impact of the endogenous neutralization activity these individuals mounted until day 9 (Figure 7C). Evaluation of the effects of S1 antibody levels in both convalescent plasma and recipients at baseline corroborated the effects of convalescent plasma on viral clearance but not recipients’ S1 antibodies, thereby reaffirming the effects of endogenous neutralizing antibodies on viral clearance (Figure 7F and Supplemental Figure 15).

## Discussion

In this study we demonstrate the capacity of convalescent plasma therapy to induce rapid viral clearance. To date, most antibody-based therapies against COVID-19, particularly convalescent plasma therapies, have shown modest benefit or no benefit (19–21). One notable exception is an outpatient trial that showed 50% reduced hospitalization rates in individuals who received high-neutralizing-content plasma (33). Highly diverse disease patterns in COVID-19 complicate the definition of a time window for treatment with neutralizing antibodies. The effect of passively administered antibodies is likely to be greatest at a stage when the patient’s own antibody response is not yet fully developed, as evidenced by trials with therapeutic SARS-CoV-2 antibody and convalescent plasma that noted benefit with administration early in the course of infection (5, 6, 11, 45, 60, 61). While reducing mortality is the primary goal of any treatment, the fact that antibody-based therapeutics can be clinically impactful at early disease stages prompted us to explore additional outcomes. Owing to its comparatively small size and specific design, the proof-of-principle study of convalescent plasma therapy for COVID-19 reported here provided the opportunity to perform comprehensive longitudinal monitoring of recipients’ virological, immunological, and disease status. In conjunction with equally comprehensive post hoc seroprofiling of donor plasma, this allowed deciphering of parameters on which the efficacy of plasma therapy depends. Our data strongly argue for an influence of convalescent plasma on rapid viral clearance by neutralizing antibodies. The fact that S1 and RBD antibody activities of different antibody isotypes were associated with rapid viral clearance supports a multifaceted action of antibodies beyond IgG-driven neutralization, including antibody effector functions such as antibody-dependent cellular cytotoxicity, and phagocytosis as well as neutralizing activity of IgA antibodies as previously suggested (62–64).

The results we present here provide key insights that may explain the apparent disparity of results of COVID-19 convalescent plasma trials. In particular, serological tests may differ in their ability to detect relevant antibody activity, as demonstrated by the failure of the Elecsys S test to repeat findings confirmed by 2 other tests, ABCORA serology and neutralization measurements. This underscores the need for standardized assay systems and the definition of appropriate correlates and surrogate markers for antibody therapies including convalescent plasma. Serological profiling is a key element to define relevant parameters particularly in the context of the dynamic interplay of recipient and donor immunity, as recently shown (65). In contrast to therapeutic antibodies, for which dosing and half-life can be optimized (17, 66), the neutralizing antibody content in convalescent plasma varies and is commonly modest (34, 35). Only small and transient increases in neutralization activity upon transfusion can be expected, given the limited plasma volumes applied — more so, as transfused antibody will be rapidly consumed by virus and eliminated with the opsonized particles.

The fact that the convalescent plasma in our study was provided during the build-up phase of the endogenous immunity may have been crucial in deciphering the effect of antibody therapy. Importantly, we found that convalescent plasma neutralizing antibodies can have an impact even in already seroconverted individuals, underlining that application for antibody therapeutics should generally not be viewed as restricted to the earliest, pre-seroconversion infection phase.

High SARS-CoV-2 viral loads correspond to disease severity (67–69). Rapid SARS-CoV-2 clearance must thus be considered as desirable, as therewith dissemination of the virus in the body will be halted and tissue damage limited. Outcome measures such as viral clearance should thus be considered to enable the detection of antibody treatment effects closer to transfusion. Yet viral clearance may not always be linked with an immediate recovery. Depending on the progression of the disease and the state of inflamed organs and tissues, recovery periods may be extended. Antibody therapeutics may thus reliably record an impact on viral clearance, but assessment of the clinical impact of stopping virus replication will remain challenging, as COVID-19 manifestation varies greatly, explaining why virus load clearance has not yet been established as a predictor of survival following convalescent plasma therapy.

Immune complexes formed by autologous spike IgG antibodies and SARS-CoV-2 have been implicated in causing lung damage (70–72), and spike IgG responses with aberrant glycosylation may in particular contribute to immunopathology (73). As Ig glycosylation patterns normalize relatively rapidly, there is likely only a short window of time during seroconversion when immunopathological spike IgG may circulate and cause severe disease. Seroconversion must thus be considered as a critical phase to target by therapeutics. In our trial, external, matured antibody was transfused at a time when the endogenous response was not yet fully complete. Antibodies in convalescent plasma are known to mature over long periods, improving their affinity and neutralization capacity (74–76). Even if low-titered they may therefore be highly effective. While immune complexes in early seroconversion may be detrimental if driven by aberrant glycosylated IgG, therapeutic antibodies and the fully matured antibody responses in convalescent plasma may substitute the endogenous response, opsonizing virus and destining it to clearance via Fc receptor– or complement receptor–bearing phagocytes.

Antibody treatments need to be carefully evaluated for the absence of disease-exacerbating effects. Excessive formation of immune complexes may advance immunopathology. Enhancement of infection may occur due to uptake of antibody-opsonized virus by Fc receptor–bearing cells or, as recently described, by triggering of the spike by a distinct type of antibodies against the N-terminal domain (77). We observed no negative impact of plasma treatment in our study. None of the measured antibody parameters was associated with slowing of viral clearance. The overall mortality rate in the 30 treated participants was low (3.3%) and well below the 9%–13% average mortality among hospitalized individuals in Switzerland (36, 37). To improve tolerability, our study design foresaw 3 smaller plasma units (200 mL) to be given on 3 consecutive days. While our study is comparatively small, we observed a safety profile of convalescent plasma that is comparable to that of plasma administered for other indications. The absence of transfusion-related adverse events supports that low-volume application together with restriction to male plasma donation should be generally considered to limit adverse events of convalescent plasma therapy.

A limitation of our study is that net effects of plasma treatment on mortality reduction could not be verified in the absence of a control group. To compensate for this limitation, the study design allowed a dose-effect analysis of neutralizing plasma antibodies by including convalescent plasma with variable levels of neutralizing antibodies. This approach allowed conclusions to be drawn about the effects of plasma with a high content of neutralizing antibodies on virus clearance. We consider several factors to be crucial to why our study showed an effect of convalescent plasma therapy that was not observed in other studies. First, although several patients already had mounted an immune response, all received the plasma therapy still at a comparatively early stage before the peak of endogenous antibody activity, thus allowing the passively administered antibodies to exert their effect. Second, the convalescent plasma was not selected for antibody content, which provided the opportunity to perform an effective component analysis to decipher whether and which antibody parameters in donor plasma have an impact. Third, multifactorial profiling of neutralizing and binding antibody responses proved critical. Fourth, we carefully controlled for confounding factors such as comorbidities, additional treatments with steroids, and remdesivir. Fifth, our study highlights the importance of assessing the immune status of patients when analyzing SARS-CoV-2 therapies. As we show, systemic viremia is inversely related to the presence of endogenous neutralizing antibody activity and should be considered a relevant marker of immunity that is not yet mature, delayed, or impaired. Our study shows a strong effect of endogenous neutralizing antibody on viral clearance, which was most pronounced in individuals who had not developed neutralizing activity at baseline. Taken together, these data argue that beyond antibody-based interventions, SARS-CoV-2 therapeutics in general must be evaluated in the context of the dynamic state of the endogenous response, as both evolving endogenous immunity and the therapeutic agent will have an impact on viral clearance.

## Methods

### Trial design.

The study was initiated in April 2020 and completed in November 2020 prior to availability of vaccines and monoclonal antibody therapeutics in Switzerland. Classification as a first-in-human trial under category C excluded formation of a placebo arm. The study was accordingly designed as a phase I, open-label, nonrandomized, single-center clinical trial, to evaluate the safety (primary outcome) and potential efficacy (secondary outcome) of SARS-CoV-2 convalescent plasma in COVID-19 disease (Figure 1A and Supplemental Tables 1–4), and focused on individuals with advanced COVID-19 disease that required hospitalization but not intensive care. Patients received 3 units of single-donor plasma donation (200 mL per unit) on 3 consecutive days, followed by an observation period of 70 days, during which clinical and laboratory parameters were monitored. Data collection and monitoring were coordinated by the Clinical Trials Center located at the trial site (University Hospital Zurich) and included an Internet-based secure database, secuTrial, for data and query management, monitoring, reporting, and coding. An independent data monitoring committee received weekly reports to assess safety and decide on continuation or termination of the trial.

### Study participants: inclusion and exclusion criteria.

Patients hospitalized with COVID-19 were recruited. No time limit after SARS-CoV-2 diagnosis or onset of symptoms was set for inclusion. Inclusion criteria were signed informed consent; COVID-19 diagnosis based on SARS-CoV-2 reverse transcriptase PCR (RT-PCR) testing; and (a) age at least 50 years and presence of at least 1 of the following risk factors for a poor outcome: preexisting cardiovascular disease, diabetic disease, chronic obstructive pulmonary disease, chronic liver disease, chronic renal failure, immunodeficiency, immunosuppression, and neoplastic disease; (b) age at least 18 years and immunosuppression or cancer; or (c) age at least 18 years and presence of at least 1 of the following signs of severe COVID-19: peripheral oxygen saturation (SpO_2_) ≤ 94% on room air, O_2_ supplementation, and typical changes on chest x-ray and/or lung CT scan. COVID-19–related exclusion criteria were (a) life-threatening COVID-19 defined as hospitalization in the intensive care unit or mechanical ventilation; (b) signs of acute respiratory distress syndrome (ARDS); and (c) cytokine release syndrome. Patients with a known history of IgA deficiency were also excluded from the study.

### SARS-CoV-2 convalescent plasma.

Plasma donor screening and selection were based on the following criteria: male, aged above 18 years, fulfilling Swiss criteria for blood donation (78, 79), recovered from RT-PCR–confirmed SARS-CoV-2 infection, 2 negative RT-PCR SARS-CoV-2 test results from NPS (at least 24 hours apart) before plasmapheresis. According to Swiss regulations in transfusion medicine (78), only male donors were allowed, in order to exclude transfusion-related acute lung injury (TRALI) reaction after transfusion of plasma obtained from female donors. Routine plasma collection procedures via plasmapheresis technology, processed and pathogen-inactivated using INTERCEPT technology according to standard operating procedures as approved by Swissmedic (80–84), were applied. Labeling of final product and release for transfusion were compliant with the requirements of the Federal Act on Medicinal Products and Medical Devices, Swissmedic, and the prescription of Blutspende Schweiz. Each plasma donation was split into 3 aliquots containing 200 mL. The plasma products were immediately frozen (fresh-frozen plasma [FFP]) and thawed upon use. In total, 75 plasma donations were collected between April and November 2020 to ascertain the availability of ABO-compatible plasma.

### Convalescent plasma transfusion.

Three units of 200 mL of convalescent plasma (FFP) of a single donor were transfused to one ABO-compatible recipient on 3 consecutive days (days 0, 1, 2; Figure 1A). Recipients had to have no signs of circulatory instability nor ARDS before transfusion. Convalescent plasma transfusion was administered at a rate of 100 mL/h. One gram paracetamol and 1 mg clemastine were administered as premedication 1 hour before convalescent plasma transfusion. After the treatment period, plasma recipients were followed up for 10 weeks, with safety visits performed 2 and 7 days as well as 3 and 10 weeks after the last plasma donation (day 2).

### Standard COVID-19 treatment.

In addition to the convalescent plasma therapy, patients received the standard treatment for COVID-19 recommended at the time. Treatment consisted of symptom control and supportive care and was continuously adjusted to guidelines of the Swiss Society of Infectious Diseases (85). Oxygen therapy was supplied per requirement. Trial participants had access to all upcoming novel COVID-19 treatment options during the trial. Depending on the clinical indication, antiviral drugs (remdesivir) and steroids (dexamethasone) were provided in addition to individually required medications for non–COVID-19–related causes including antibacterial therapy.

### Monitoring of transfusion-related adverse events.

Patients were monitored by expert hematologists for occurrence of adverse events due to plasma therapy, including TRALI, anaphylactic reactions, hemolytic reactions, and transfusion-associated circulatory overload.

### Clinical laboratory parameters.

To assess safety and beneficial effects of convalescent plasma therapy as defined in the trial’s outcome measures (Supplemental Table 4), an extended evaluation of clinical parameters was conducted based on routine diagnostic analyses from chemistry, hematology, immunology, microbiology, and virology (Supplemental Table 3). SARS-CoV-2–specific analyses are detailed below.

### Assessing overall clinical status.

We included a 7-category “pulmonary” ordinal outcome used in the TICO trial as a measure to rate overall clinical improvement as previously described (45). This pulmonary outcome allows a longitudinal assessment of disease stage. We did not focus our analyses on length of hospitalization owing to local circumstances, as during the early phase of the pandemic, release from the hospital in our trial depended not only on the health status but also on de-isolation rules that were adapted over time by authorities.

### Quantitative SARS-CoV-2 RT-PCR.

NPSs and plasma were analyzed for SARS-CoV-2 by RT-PCR using the cobas SARS-CoV-2 IVD test (Roche Diagnostics GmbH) as previously described (86). Samples are recorded negative when both E-gene and ORF-1 are not detected. Ct values are recorded for positive RT-PCR results.

### ABCORA 2 multiplex SARS-CoV-2 serology profiling.

Seroreactivity to SARS-CoV-2 was profiled in EDTA plasma (1:100 diluted) using the in-house-developed multiplex bead assay ABCORA 2 (87). The assay measures IgG, IgA, and IgM reactivity to 4 SARS-CoV-2 antigens, RBD, S1, S2, and N (12 SARS-CoV-2 parameters), in addition to IgG, IgA, and IgM reactivity to S1 of HCoV-HKU1. Raw median fluorescence intensity (MFI) values were corrected for background binding to empty beads and recorded as MFI-FOE (fold over empty beads; ref. 87). Using predefined thresholds for positivity for each of the 12 measured SARS-CoV-2 parameters (87), signal over cutoff (SOC) values (MFI-FOE/threshold) were derived. SOC values greater than 1 denote positive reactivity; SOC less than 1 denotes negative reactivity. SARS-CoV-2–positive plasma reactivity was defined using the ABCORA 2.3 computational approach, achieving 99.07% specificity and 94.29% sensitivity (87). Antibody binding titers were defined by serial plasma dilution and recorded as 50% effective titer concentrations (EC_50_) using a 4-parameter logistic curve [*y* = bottom + (top – bottom)/(1 + 10^(logEC50^^–^^x)^) × Hill slope] as described previously (87).

### Elecsys Anti-SARS-CoV-2 S assay.

Donor plasma was evaluated with the Elecsys Anti-SARS-CoV-2 S assay (Roche Diagnostics GmbH; referred to here as Elecsys S), a quantitative electro-chemiluminescence immunoassay that detects total antibodies against the SARS-CoV-2 S protein RBD, according to the manufacturer’s instructions. Results are recorded in U/mL. Values ≥0.80 U/mL are recorded as positive for anti–SARS-CoV-2 S antibodies.

### SARS-CoV-2 pseudo-neutralization assay.

SARS-CoV-2 plasma neutralization activity against Wuhan-Hu-1 pseudoviruses was assessed as previously described (87, 88) using the env-inactivated HIV-1 reporter construct pHIV-1NL4-3 ΔEnv-NanoLuc (pHIV-1Nanoluc) and HT1080/ACE2cl.14 cells (both provided by P. Bieniasz, Rockefeller University, New York, New York, USA) and the C-terminal truncated SARS-CoV-2 spike expression plasmid P_CoV2_Wuhan. Pseudotypes were produced in HEK293T cells (American Type Culture Collection). Plasma neutralization titers causing 50% reduction in viral infectivity (NT_50_) in comparison with controls without plasma were calculated by fitting of a sigmoid dose-response curve (variable slope), using GraphPad Prism with constraints (bottom = 0, top *=* 100). If 50% inhibition was not achieved at the lowest plasma dilution of 1:100, a “less than” value was recorded. All measurements were conducted in duplicate.

### SARS-CoV-2 whole-genome sequencing.

During the trial period (April–November 2020), SARS-CoV-2 lineages derived from B.1, harboring D614G but otherwise closely related to the original Wuhan-Hu-1 strain (MN908947.3), were prevalent in Switzerland (GISAID; www.gisaid). SARS-CoV-2 whole-genome sequencing was performed according to the nCoV-2019 sequencing protocol v3 (LoCost) V.3 (89, 90). Briefly, total nucleic acids were extracted, followed by reverse transcription with random hexamers using LunaScript RT SuperMix Kit (New England Biolabs). The generated cDNA was used as input for 2 pools of overlapping PCR reactions (~400 nt each) spanning the viral genome using Q5 Hot Start High-Fidelity 2X Master Mix (New England Biolabs). Amplicons were pooled per patient before NexteraXT library preparation and sequencing on an Illumina MiSeq for 1 × 151 cycles. To generate SARS-CoV-2 consensus sequences, reads were iteratively aligned using SmaltAlign (91).

### Statistics.

Statistical analyses were performed in R (version 4.0.5) and plotted using the ggplot2 package (92). All reported statistical analyses are of an explorative, descriptive nature. We therefore opted not to adjust for multiple testing. Completion of plasma therapy (day 2, after the third dose) was defined as end of treatment. To assess benefits of treatment, laboratory and respiratory outcomes were compared between baseline and follow-up visits using a 2-sided, paired *t* test. Plasma recipients with missing values either at baseline or after therapy were not considered in this analysis.

Differences in clinical, laboratory, and immunological parameters in recipient subgroups were analyzed by 2-sided *t* test. Differences in times to viral clearance according to given subgroups were assessed either by Kaplan-Meier analysis or by interval-censored parametric survival models. In the Kaplan-Meier analyses, time to viral clearance was defined as the first negative PCR test (no Ct or Ct 45) that was not followed by any positive test. The parametric survival model assumed a γ-distributed time to clearance and used the last positive and first negative SARS-CoV-2 RT-PCR tests in NPS to define the interval in which the virus was cleared. The difference in time to viral clearance between the 2 groups was modeled assuming proportional hazard. We adjusted these analyses for 2 potential confounders: baseline viral load and the presence of any comorbidity. Finally, we determined the impact of the donor plasma on virus decay using censored regression.

### Study approval.

The study (ClinicalTrials.gov NCT04869072) was approved by the local ethics committee Kantonale Ethikkomission Kanton Zürich (KEK) of the Canton of Zurich, Switzerland (BASEC 2020-00787). Written informed consent was obtained from all study participants and convalescent plasma donors. The study was classified under category C and approved by the Swiss authorities (Swissmedic 2020TpP1004; www.swissmedic.ch).

## Author contributions

HFG, MGM, AT, and BMF conceptualized and designed the study. MM, DLB, EW, RK, HFG, ES, BMF, and MGM conducted clinical investigations. IAA, SE, AA, MH, SS, MZ, MS, JW, PR, JB, and VK carried out the laboratory-based investigations. AT, RDK, and IAA developed the methodology. AH, RDK, and CP performed statistical analyses. MS and IAA created the figures. HFG, MGM, AT, and BMF acquired funding. MM, EW, and RK monitored the study. AT, IAA, RDK, MGM, and BMF supervised the study. AT, IAA, and MM wrote the original draft of the manuscript. AT, IAA, AH, RK, MS, DLB, SE, AA, JW, PR, ES, CP, EW, JB, VK, MH, MZ, SS, BMF, RDK, HFG, and MGM wrote, reviewed, and edited the manuscript. The order of first authors was based on their sequential roles in the study: MM, clincial lead; IAA, laboratory analysis; AH, statistics.

## Supplementary Material

Supplemental data

## Figures and Tables

**Table 2:**
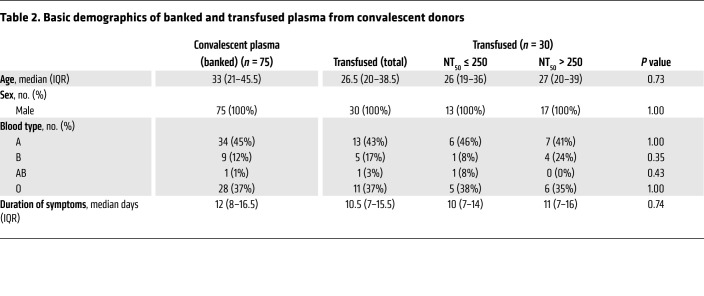
Basic demographics of banked and transfused plasma from convalescent donors

**Table 1:**
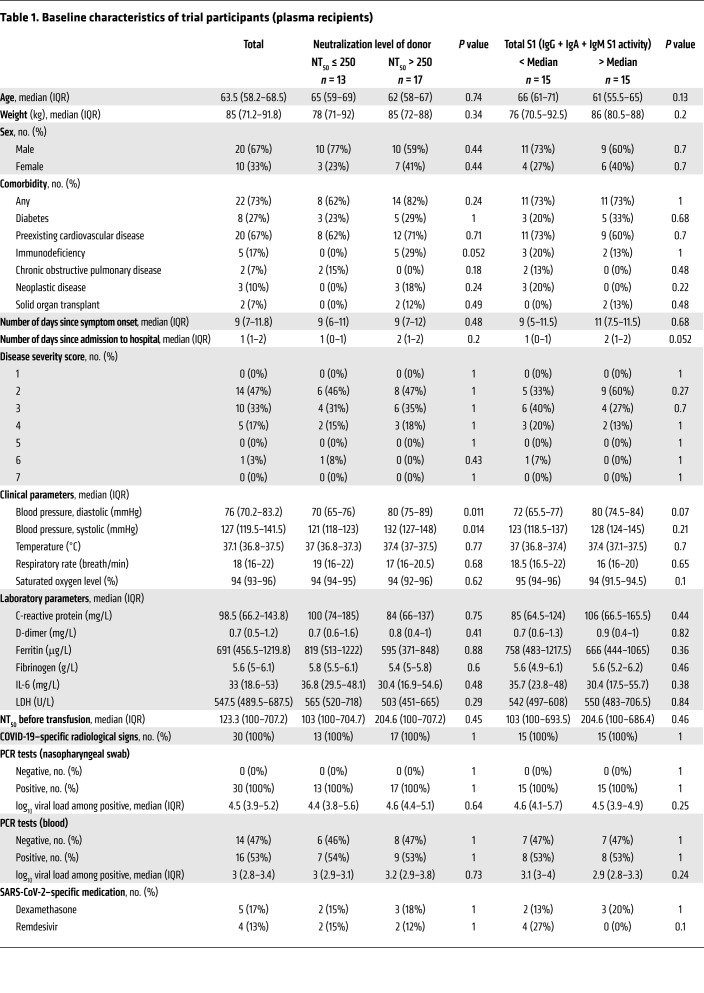
Baseline characteristics of trial participants (plasma recipients)

## Version 1 04/28/2022

In-Press Preview

## Version 2 06/15/2022

Electronic publication
