# Supplementary material for: Antibodies from convalescent plasma promote SARS-CoV-2 clearance in individuals with and without endogenous antibody response
Source: J Clin Invest. 2022 Jun 15;132(12):e158190. doi: 10.1172/JCI158190 (PMC9197521; doi:10.1172/JCI158190)
Supplement: Supplemental data [file jci-132-158190-s219.pdf]

Supplementary Materials for

Antibodies from convalescent plasma promote SARS-CoV-2 clearance in individuals with and without endogenous antibody response

Marconato, Abela, Hauser et al.

Summary of supplementary tables and figures

Table S1: Study schedule

Table S2: Inclusion/Exclusion criteria of trial participants (plasma recipients)

Table S3: Monitored parameters

Table S4: Primary and secondary outcomes

Table S5: Clinical and immunological characteristics of recipients

Table S6: Safety outcomes

Table S7: Severe adverse events (SAE)

Table S8: Pulmonary ordinal outcome

Table S9: Severity outcome of trial participants assessed by pulmonary ordinal outcome

Table S10: Laboratory measures and clinical parameters

Table S11: Hazard ratios of time to viral clearance for binding and neutralizing parameters, presence of comorbidity and baseline viral load in nasopharyngeal swab.

Table S12: Summary of recipients' characteristics stratified by baseline endogenous neutralization

Fig. S1: Phylogenetic analysis of the SARS-CoV-2 isolates from trial participants

Fig. S2: Longitudinal assessment of primary and secondary outcomes

Fig. S3: No impact of neutralization activity on hospital release

Fig. S4: Sensitivity analysis for impact of plasma on viral clearance

Fig. S5: Spike specific binding and neutralizing antibodies are linked with rapid virus clearance

Fig. S6: Sensitivity analysis for impact of binding and neutralizing antibodies on viral clearance (excluding remdesivir treated patients)

Fig. S7: Sensitivity analysis for impact of binding and neutralizing antibodies on viral clearance (excluding dexamethasone treated patients)

Fig. S8: Sensitivity analysis for impact of binding and neutralizing antibodies on viral clearance (excluding dexamethasone, remdesivir treated patients, or patients incapable of mounting an antibody response.)

Fig. S9: No impact on viral clearance when stratifying based on the Elecsys S assay

Fig. S10: SARS-CoV-2 antibody reactivities are correlated within antibody classes

Fig. S11: High neutralizing plasma leads to faster virus decay in nasopharyngeal swabs

Fig. S12: Evolution of SARS-CoV-2 antibodies in trial participants

Fig. S13: Antibody response over time in the three trial participants incapable of mounting antibody response

Fig. S14: Sensitivity survival analysis adjusting for the impact of endogenous neutralization on virus clearance

Fig. S15: Sensitivity survival analysis adjusting for the impact of endogenous binding antibodies on virus clearance.

Table S1: Study schedule

**Study period**	**Screening**	**Treatment period**					**Safety follow-up**	
**Visit**	**1**	**2**	**3**	**4**	**5**	**6**	**7**	**8**
**Time** (days)	**0**	**0**	**1**	**2**	**4**	**9**	**23+/-1**	**72+/-1**
Patient information and informed consent	**x**							
In-/exclusion criteria	**x**	**x**						
Medical history and demographic data	**x**							
Documentation of concomitant drugs	**x**	**x**	**x**	**x**	**x**	**x**	**x**	**x**
Body weight/height	**x**	**x**					**x**	**x**
Physical examination	**x**						**x**	**x**
Vital signs	**x**	**x**	**x**	**x**	**x**	**x**	**x**	**x**
**Screening laboratory measurements**	**x**							
**Blood typing**	**x**							
**Safety laboratory tests**	**x**	**x**	**x**	**x**	**x**	**x**	**x**	**x**
**Laboratory measures**	**x**	**x**	**x**	**x**	**x**	**x**	**x**	**x**
Urine pregnancy test	**x**							**x**
Samples for biobank (optional)	**x**				**x**	**x**	**x**	**x**
Administer study medication		**x**	**x**	**x**				
Severe adverse/adverse events	**x**	**x**	**x**	**x**	**x**	**x**	**x**	**x**

Table S2: **Inclusion/exclusion criteria of trial participants**

**Inclusion** criteria:	**Exclusion** criteria:
A) Proven SARS-CoV-2 by PCR and hospitalization for COVID-19 in combination with either (1) or (2):<br>(1) Age ≥ 50<br><br>AND (at least one):<br>● Pre-existing cardiovascular disease<br>● Diabetic disease<br>● Immunodeficiency/immunosuppression<br>● Neoplastic disease<br>● COPD or chronic liver disease or chronic renal failure<br><br>(2) Age ≥ 18<br><br>AND (at least one):<br>● SpO2 ≤ 94% on room air or requiring supplemental oxygen at screening<br>● Typical changes on chest x-ray and/or lung-CT scan<br>● Immunosuppression or neoplastic disease<br><br>B) Written informed consent as documented by signature (Appendix Informed Consent Form) of the patient or, in case of inability, of the next relative/care-taking person. In the latter case, an independent doctor will also be involved and her/his signature will be required in order to enrol the patient.	1. Contraindications to the class of drugs under study, e.g. known hypersensitivity or allergy to class of drugs or the investigational product (FFP)<br>2. Known IgA deficiency<br>3. Cytokine Release Syndrome grade ≥ 3<br>4. Acute respiratory distress syndrome (ARDS)<br>5. Patients already hospitalized in intensive care unit and/or already receiving mechanical ventilation<br>6. Known or suspected non-compliance, drug or alcohol abuse<br>7. Previous enrolment into the current study<br>8. Enrolment of the investigator, his/her family members, employees and other dependent persons<br>9. Women who are pregnant or breast feeding<br>10. Intention to become pregnant during the course of the study<br>11. Lack of safe contraception, defined as: Female participants of childbearing potential, not using and not willing to continue using a medically reliable method of contraception for the entire study duration, such as oral, injectable, or implantable contraceptives, or intrauterine contraceptive devices, or who are not using any other method considered sufficiently reliable by the investigator in individual cases. Please note that female participants who are surgically sterilised/hysterectomised or post-menopausal for longer than 2 years are not considered as being of child bearing potential

Table S3: Monitored parameters

Monitored clinical parameter and laboratory measures	Baseline	Treatment period	Follow-up period
	Day 0	Day 0-2, 9	23 +/-1, 72+/-1
**Clinical parameters:**			
Blood pressure (mmHg), Heart rate (bpm*)<br>Respiratory rate (bpm**)<br>Body temperature (°C, ear)<br>Oxygen saturation (SpO2 (%))	X	X	X
**Laboratory measures:**			
Blood count (WBC subsets), INR<br>CRP (mg/L), Ferritin (ug/L), LDH (unit/L)<br>D-dimer (mg/L), Fibrinogen (g/L), IL-6<br>Na, K, Ca, Serum Creatinine (mg/dL)<br>Liver enzymes: AST (SGOT) (U/L), ALT (SGPT) (U/L), γGT, Troponin I, Myoglobin<br>Rapid urine test	X	X	X
25-OH Vitamin, Albumin<br>Urin sediment<br>IgG, IgG subclasses, IgA, IgM<br>PT, PTT	X		
SARS-CoV-2 antibody binding response (IgM, IgG, IgA response against S1, S2, RBD, N)	X		X
SARS-CoV-2 neutralizing antibody binding titers (NT50)	X		
PCR for SARS-CoV-2 on nasal swab, plasma, BAL	X	X	X

*beat per minute, **breaths per minute,

Table S4. Primary and secondary outcomes

**Primary outcomes:**
**(A) Safety measures**
**1. Assessment for**<br>• Transfusion related acute lung injury (TRALI)<br>• Anaphylactic reactions<br>• Hemolytic reactions<br>**2. Clinical monitoring and measurement of laboratory parameters**<br>• During treatment episode: Day 0,1,2<br>• Follow up : Day 4, Day 9, Day 23 +/- 1 and Day 72 +/- 1
**(B) Efficacy measures**
**1. Improvement of respiration:**<br>• Respiratory rate (breaths per minute, bpm), Oxygen saturation (SpO2 (%))<br>**2. Improvement of laboratory measures:**<br>• CRP (mg/L),<br>• Ferritin (ug/L)<br>• IL-6 (mg/L)<br>• D-dimer (mg/L)<br>• Fibrinogen (g/L)<br>• LDH (unit/L)<br>**3. Reduction of hospitalization duration**<br>**4. Prevention of ICU-admission or shortening of ICU-hospitalization**
**Secondary outcomes:**
**1. Characterize humoral response (binding and neutralization) against SARS-CoV-2**<br>**2. Longitudinally assess viral shedding in nasopharyngeal swabs and blood samples**

Table S5. Clinical and immunological characteristics of recipients

ID	Gender	Comorbidity	Comorbidity type	Immune suppression	Impaired antibody response	Endogenous SARS-CoV-2 neutralization at baseline	Log10 viral load NPS at baseline	Viral suppression blood at baseline	Remdesivir treatment	Steroid treatment	Convalescent plasma (NT50 low/high)	Clinical outcome at study completion (day 72)	Infecting strain
1	Female	Yes	Solid organ transplant (kidney)	yes	No	Yes	4.7	Yes	No	No	high	1	B.1.1
2	Male	Yes	Cardiovascular disease	No	Yes	No	3.93	Yes	No	No	high	2	-
3	Female	Yes	Cardiovascular disease, Diabetic disease	No	No	Yes	4.48	Yes	No	No	high	1	-
4	Male	Yes	Cardiovascular disease, Neoplastic disease (Diffuse large-cell B cell NHL)	Yes	Yes	No	5.01	No	No	No	high	1	B.1
5	Male	Yes	Cardiovascular disease	No	No	Yes	4.34	Yes	No	No	low	1	B.1.1
6	Female	No		No	No	Yes	3.54	Yes	No	No	low	1	C.35
7	Male	Yes	Cardiovascular disease	No	No	Yes	4.65	Yes	No	No	low	1	C.35
8	Male	No		No	No	Yes	4.62	Yes	No	No	high	1	B.1.1.70
9	Female	No		No	No	Yes	5.32	No	No	No	high	1	B.1.1
10	Female	Yes	Cardiovascular disease, Diabetic disease	no	No	No	2.81	No	No	Yes	high	1	B.1.1
11	Male	Yes	Cardiovascular disease, Neoplastic disease (plasma cell leukaemia)	Yes	No	Yes	4.43	Yes	Yes	No	high	1	B.1.1
12	Male	Yes	Cardiovascular disease, Diabetic disease, COPD	No	No	No	5.86	No	No	No	low	1	B.1.1.70
13	Male	Yes	Cardiovascular disease	No	No	Yes	4.79	Yes	No	No	low	1	B.1.1.70
14	Male	No		No	No	Yes	3.77	No	No	No	high	1	C.35
15	Female	Yes	Cardiovascular disease, Neoplastic disease (CLL)	Yes	Yes	No	9.85	No	Yes	No	high	7	C.35
16	Male	Yes	Cardiovascular disease, Diabetic disease	No	No	Yes	3.91	No	No	No	low	1	B.1.1.47
17	Female	Yes	Cardiovascular disease, Diabetic disease	No	No	Yes	3.79	Yes	No	No	low	1	B.1.1.47
18	Female	No		No	No	No	5.8	No	Yes	No	low	2	B.1.1.70
19	Male	Yes	Cardiovascular disease, COPD	No	No	No	6.42	No	No	No	low	1	C.35
20	Male	No		No	No	No	3.85	No	No	No	low	1	C.35
21	Female	Yes	Cardiovascular disease, Diabetic disease	No	No	Na	4.39	Yes	No	No	high	1	B.1.1.58
22	Male	Yes	Cardiovascular disease, Chronic renal failure	No	No	Yes	5.15	Yes	No	No	high	1	B.1.1.70
23	Male	Yes	Cardiovascular disease, Diabetic disease	No	No	No	3.15	No	No	No	high	1	
24	Male	Yes	Cardiovascular disease	No	No	Yes	6.97	No	No	Yes	high	2	B.1.1
25	Male	Yes	Cardiovascular disease	No	No	Yes	4.4	No	No	No	high	1	B.1.1.39
26	Male	Yes	Cardiovascular disease	No	No	No	5.56	No	No	Yes	low	2	B.1.1.39
27	Male	No		No	No	Yes	3.57	Yes	No	No	low	1	-
28	Female	Yes	Cardiovascular disease, Diabetic disease	No	No	Yes	5.19	No	No	Yes	high	1	B.1
29	Male	No		No	No	No	4.35	No	Yes	Yes	low	1	-
30	Male	Yes	Solid organ transplant (kidney)	Yes	No	No	4.56	Yes	No	No	high	1	-

Table S6: **Safety outcomes**

	**Total**
**Complications of CPT therapy (safety), No (%)**	
Transfusion Related Acute Lung Injury (TRALI)	0 (0%)
Anaphylactic Reactions (AR)	0 (0%)
Hemolytic Reactions (HR)	0 (0%)
Transfusion associated circulatory overload (TACO)	0 (0%)
**Clinical and laboratory outcomes (efficacy), No (%)**	
COVID-19 related death during the study	0 (0%)
Hospitalization in ICU	2/30 (6.67%)
Mechanical ventilation	2/30 (6.67%)

Table S7: Severe adverse events (SAE)

ID	Gender	Description of event	Description of intervention	Outcome	Comments, if relevant
3	Female	NSTEMI	No specific treatment needed	improved	Possible correlation with COVID-19
10	Female	Hospital acquired pneumonia (HAP)	antibiotics	resolved	COVID-19 resolved, HAP
15	Female	Hospital acquired pneumonia (HAP)	antibiotics	death	COVID-19 resolved, HAP
17	Female	Pulmonary Embolism	Anticoagulation with heparin and liquemin	resolved	Complication of COVID-19
18	Female	Respiratory insufficiency due to COVID-19	Patient transferred to the ICU, mechanical ventilation needed	resolved	-
20	Male	Arthritis (right lateral malleolus)	Local corticosteroids	resolved	-
26	Male	Respiratory insufficiency due to COVID-19	Patient transferred to the ICU mechanical ventilation needed	resolved	

Table S8: **Pulmonary ordinal outcome**

1= Can independently undertake usual activities with minimal or no symptoms
2= Symptomatic and currently unable to independently undertake usual activities but no need of supplemental oxygen (or not above premorbid requirements)
3= Supplemental oxygen (<4 liters/min, or <4 liters/min above premorbid requirements)
4= Supplemental oxygen (≥4 liters/min, or ≥4 liters/min above premorbid requirements, but not high-flow oxygen)
5= Non-invasive ventilation or high-flow oxygen
6=Invasive ventilation, extracorporeal membrane oxygenation (ECMO), mechanical circulatory support, or new receipt of renal replacement therapy
7=Death

Table S9: **Severity outcome of study participants assessed by pulmonary ordinal outcome**

	Before/during therapy			Follow-up visits		Safety visits	
	Day 0	Day 1	Day 2	Day 4	Day 9	Day 23 +/- 1	Day 72 +/- 1
**Ordinal scale**							
1	0 (0%)	0 (0%)	0 (0%)	1 (3%)	12 (40%)	21 (70%)	25 (83%)
2	14 (47%)	16 (53%)	14 (47%)	16 (53%)	12 (40%)	6 (20%)	4 (13%)
3	10 (33%)	9 (30%)	10 (33%)	9 (30%)	3 (10%)	1 (3%)	0 (0%)
4	5 (17%)	4 (13%)	4 (13%)	2 (7%)	1 (3%)	0 (0%)	0 (0%)
5	0 (0%)	0 (0%)	0 (0%)	0 (0%)	0 (0%)	0 (0%)	0 (0%)
6	1 (3%)	1 (3%)	2 (7%)	2 (7%)	2 (7%)	1 (3%)	0 (0%)
7	0 (0%)	0 (0%)	0 (0%)	0 (0%)	0 (0%)	1 (3%)	1 (3%)

Table S10: **Longitudinal laboratory measures and clinical parameters of study participants**

	Day 0	Day 1		Day2		Day 4		Day 9		Day 23 +/- 1		Day 72 +/- 1	
**Clinical parameters**	median (IQR)	median (IQR)	p-value	median (IQR)	p-value	median (IQR)	p-value	median (IQR)	p-value	median (IQR)	p-value	median (IQR)	p-value
Respiratory rate	18 (16-22)	18 (16.2-24)	0.56	18 (16.2-23.8)	0.4	18 (14-22)	0.12	14 (14-17.5)	0.0015	14 (14-14)	<0.001	14 (12-14)	<0.001
Oxygen saturation level	94 (93-96)	94 (93-95)	0.56	94 (92.2-96)	0.45	95 (94-96)	0.36	97 (96-98)	<0.001	97 (96-98)	<0.001	97 (97-98)	<0.001
Temperature (in °C)	37.1 (36.8-37.5)	37.1 (36.8-37.6)	0.85	37.1 (36.8-37.4)	0.85	36.9 (36.6-37.2)	0.043	36.8 (36.4-37)	0.028	36.8 (36.5-36.9)	<0.001	36.6 (36.5-36.8)	<0.001
**Laboratory parameters**	median (IQR)	median (IQR)	p-value	median (IQR)	p-value	median (IQR)	p-value	median (IQR)	p-value	median (IQR)	p-value	median (IQR)	p-value
CRP	98.5 (66.2-143.8)	92.5 (53.8-117.2)	0.11	76.5 (42.2-99.2)	0.0038	40 (19.2-58)	<0.001	6.4 (3.5-18.8)	<0.001	1.4 (0.8-4.9)	<0.001	1.5 (0.6-2.6)	<0.001
D-dimer	0.705 (0.5-1.2)	0.64 (0.4-1)	0.47	0.755 (0.5-1.1)	0.72	0.75 (0.5-1.1)	0.73	0.61 (0.4-1.4)	0.46	0.53 (0.4-0.8)	0.3	0.365 (0.3-0.5)	0.012
Ferritin	691 (456.5-1219.8)	716 (425.8-1152)	0.86	768 (486-1389)	0.92	734.5 (327.2-1278.2)	0.75	454.5 (249.8-592)	0.04	234 (122-311.5)	<0.001	74 (43-170.2)	<0.001
Fibrinogen	5.6 (5-6.1)	5.1 (4.7-5.5)	0.22	5.2 (4.7-6)	0.31	4.9 (4.3-5.8)	0.0062	4.1 (3.3-4.6)	<0.001	3.3 (2.9-3.8)	<0.001	3 (2.7-3.5)	<0.001
IL-6	33.05 (18.6-53)	35.7 (21.2-67.3)	0.7	31 (14-49.7)	0.59	10.6 (4.8-26.6)	0.22	4.7 (3.3-10.3)	0.033	2.7 (1.9-4.1)	0.027	2.4 (1.9-4)	0.036
LDH	547.5 (489.5-687.5)	592 (522.8-713.8)	0.64	587 (504-703)	0.83	550 (459-696)	0.74	477 (410.8-545.8)	0.01	368 (326-458)	<0.001	401.5 (358.8-439.8)	<0.001

Table S11: Hazard ratios of time to viral clearance for binding and neutralizing parameters, presence of comorbidity and baseline viral load in nasopharyngeal swab. Estimates for both univariable and multivariable models are given.

Antibody	Antigen	Binding/neutralizing antibody				Baseline viral load				Presence of comorbidity			
		Univariate model		Multivariate model		Univariate model		Multivariate model		Univariate model		Multivariate model	
		HR (95% CI)	p-value	HR (95% CI)	p-value	HR (95% CI)	p-value	HR (95% CI)	p-value	HR (95% CI)	p-value	HR (95% CI)	p-value
Neutralization		2.4 [1,5.9]	0.05	3 [1.1,8.1]	0.026	0.53 [0.34,0.84]	0.0064	0.52 [0.32,0.84]	0.008	0.56 [0.22,1.4]	0.23	0.62 [0.22,1.8]	0.36
Roche		2.5 [1,6]	0.048	2.1 [0.75,5.6]	0.16	0.53 [0.34,0.84]	0.0064	0.46 [0.27,0.78]	0.0036	0.56 [0.22,1.4]	0.23	0.91 [0.33,2.5]	0.85
IgG	RBD	2.7 [1.1,6.5]	0.032	2.4 [0.93,6.2]	0.071	0.53 [0.34,0.84]	0.0064	0.5 [0.31,0.82]	0.0055	0.56 [0.22,1.4]	0.23	0.79 [0.28,2.2]	0.66
IgG	S1	1.5 [0.62,3.5]	0.38	3.5 [1.3,9.3]	0.013	0.53 [0.34,0.84]	0.0064	0.5 [0.3,0.82]	0.0061	0.56 [0.22,1.4]	0.23	0.8 [0.28,2.3]	0.68
IgG	S2	1.5 [0.63,3.6]	0.35	2.9 [1,7.9]	0.042	0.53 [0.34,0.84]	0.0064	0.46 [0.28,0.76]	0.0024	0.56 [0.22,1.4]	0.23	0.88 [0.32,2.5]	0.81
IgG	N	1.6 [0.66,3.8]	0.3	1.1 [0.44,2.6]	0.89	0.53 [0.34,0.84]	0.0064	0.54 [0.34,0.87]	0.012	0.56 [0.22,1.4]	0.23	0.92 [0.33,2.6]	0.88
IgG	RBD+S1+S2+N	1.7 [0.72,4.1]	0.22	2.5 [0.96,6.4]	0.062	0.53 [0.34,0.84]	0.0064	0.49 [0.3,0.8]	0.0039	0.56 [0.22,1.4]	0.23	0.85 [0.31,2.3]	0.76
IgA	RBD	2.5 [1,6]	0.048	6.1 [1.9,20]	0.0027	0.53 [0.34,0.84]	0.0064	0.4 [0.23,0.7]	0.0011	0.56 [0.22,1.4]	0.23	0.84 [0.3,2.4]	0.74
IgA	S1	1.7 [0.71,4.1]	0.24	6.1 [1.9,20]	0.0027	0.53 [0.34,0.84]	0.0064	0.4 [0.23,0.7]	0.0011	0.56 [0.22,1.4]	0.23	0.84 [0.3,2.4]	0.74
IgA	S2	0.48 [0.19,1.2]	0.1	4 [1.4,12]	0.011	0.53 [0.34,0.84]	0.0064	0.41 [0.24,0.69]	0.00096	0.56 [0.22,1.4]	0.23	0.73 [0.26,2.1]	0.55
IgA	N	2.2 [0.88,5.2]	0.092	5 [1.6,15]	0.0049	0.53 [0.34,0.84]	0.0064	0.4 [0.23,0.7]	0.0015	0.56 [0.22,1.4]	0.23	0.83 [0.3,2.3]	0.72
IgA	RBD+S1+S2+N	0.95 [0.4,2.3]	0.92	6.1 [1.9,20]	0.0027	0.53 [0.34,0.84]	0.0064	0.4 [0.23,0.7]	0.0011	0.56 [0.22,1.4]	0.23	0.84 [0.3,2.4]	0.74
IgM	RBD	1.7 [0.71,4.1]	0.23	2.5 [0.96,6.3]	0.061	0.53 [0.34,0.84]	0.0064	0.5 [0.31,0.83]	0.0064	0.56 [0.22,1.4]	0.23	0.73 [0.26,2]	0.55
IgM	S1	2.5 [1,6]	0.048	4.1 [1.5,11]	0.0063	0.53 [0.34,0.84]	0.0064	0.45 [0.27,0.77]	0.0031	0.56 [0.22,1.4]	0.23	0.75 [0.27,2.1]	0.58
IgM	S2	1.7 [0.72,4.1]	0.22	2.7 [1,7.2]	0.049	0.53 [0.34,0.84]	0.0064	0.46 [0.27,0.76]	0.0026	0.56 [0.22,1.4]	0.23	0.88 [0.32,2.5]	0.81
IgM	N	2.8 [1.1,6.7]	0.027	0.68 [0.26,1.8]	0.45	0.53 [0.34,0.84]	0.0064	0.57 [0.35,0.94]	0.028	0.56 [0.22,1.4]	0.23	0.98 [0.35,2.8]	0.98
IgM	RBD+S1+S2+N	1.6 [0.69,4]	0.26	3.4 [1.3,9.5]	0.017	0.53 [0.34,0.84]	0.0064	0.43 [0.25,0.72]	0.0016	0.56 [0.22,1.4]	0.23	0.97 [0.35,2.7]	0.96

Table S12: Summary of recipients' characteristics stratified by baseline endogenous neutralization

	Total	Pre-transfusion neutralization level of recipient		p-value
		NT50=100	NT50>100	
		N=12	N=17	
**Age**, median (IQR)	63.5 (58.2-68.5)	69.5 (62.2-74.5)	61 (57-66)	0.12
**Weight** (kg), median (IQR)	85 (71.2-91.8)	86.5 (66-94.2)	83 (72-88)	0.46
**Sex**, No. (%)				
Male	20 (67%)	9 (75%)	11 (65%)	0.39
Female	10 (33%)	3 (25%)	6 (35%)	0.39
**Comorbidity**, No. (%)				
Any	22 (73%)	9 (75%)	12 (71%)	1
Diabetes	8 (27%)	3 (25%)	4 (24%)	0.33
Pre-existing cardiovascular disease	20 (67%)	8 (67%)	11 (65%)	1
Immunodeficiency	5 (17%)	3 (25%)	2 (12%)	0.69
COPD	2 (7%)	2 (17%)	0 (0%)	0.22
Neoplastic disease	3 (10%)	2 (17%)	1 (6%)	0.6
Solid organ transplant	2 (7%)	1 (8%)	1 (6%)	1
**Number of days since symptom onset**, median (IQR)	9 (7-11.8)	7.5 (5.5-11)	11 (9-12)	0.25
**Number of days since admission to hospital**, median (IQR)	1 (1-2)	1 (0.8-2.2)	1 (1-2)	0.75
**Disease severity score**, No. (%)				
1	0 (0%)	0 (0%)	0 (0%)	1
2	14 (47%)	6 (50%)	7 (41%)	0.58
3	10 (33%)	3 (25%)	7 (41%)	0.63
4	5 (17%)	2 (17%)	3 (18%)	1
5	0 (0%)	0 (0%)	0 (0%)	1
6	1 (3%)	1 (8%)	0 (0%)	0.43
7	0 (0%)	0 (0%)	0 (0%)	1
**Clinical parameters**, median (IQR)				
Blood pressure, diastolic (mmHg)	76 (70.2-83.2)	79 (70.8-83)	74 (67-84)	0.49
Blood pressure, systolic (mmHg)	127 (119.5-141.5)	138 (118.8-154.2)	126 (121-131)	0.15
Temperature (°C)	37.1 (36.8-37.5)	37.3 (37-37.8)	37 (36.7-37.4)	0.083
Respiratory rate (breath/min)	18 (16-22)	19 (16-21)	18 (16-22)	0.9
Saturated oxygen level (%)	94 (93-96)	94 (94-96.2)	94 (93-96)	0.24
**Laboratory parameters**, median (IQR)				
CRP (mg/L)	98.5 (66.2-143.8)	77 (65.5-155.8)	99 (74-137)	0.79
D-dimer (mg/L)	0.7 (0.5-1.2)	0.7 (0.6-1.2)	0.6 (0.5-1)	0.54
Ferritin (µg/L)	691 (456.5-1219.8)	574.5 (466-926.5)	819 (461-1282)	0.34
Fibrinogen (g/L)	5.6 (5-6.1)	5 (4.6-5.7)	5.8 (5.5-6.3)	0.03
IL-6 (mg/L)	33 (18.6-53)	36.2 (28.2-46.1)	30.4 (18.2-62.3)	0.31
LDH (unit/L)	547.5 (489.5-687.5)	550.5 (500.5-642.5)	550 (489-761)	0.61
**Covid-19 specific radiological signs,** No. (%)	30(100%)	13 (100%)	17 (100%)	1
**PCR tests (nasopharyngeal swab)**				
Negative, No. (%)	0 (0%)	0 (0%)	0 (0%)	1
Positive, No. (%)	30 (100%)	12 (100%)	17 (100%)	1
Log10 viral load among positive, median (IQR)	4.5 (3.9-5.2)	4.8 (3.9-5.8)	4.5 (3.9-4.8)	0.37
**PCR tests (blood)**				
Negative, No. (%)	14 (47%)	2 (17%)	11 (65%)	0.014
Positive, No. (%)	16 (53%)	10 (83%)	6 (35%)	0.014
Log10 viral load among positive, median (IQR)	3 (2.8-3.4)	3.2 (3-3.7)	2.9 (2.8-3)	0.18
**SARS-CoV-2 specific medication**, No. (%)				
Dexamethasone	5 (17%)	3 (25%)	2 (12%)	0.69
Remdesivir	4 (13%)	3 (25%)	1 (6%)	0.37

Supplementary Figure legends

Fig. S1: Phylogenetic analysis of the SARS-CoV-2 isolates from 26 trial participants. Phylogenetic analysis of sequences derived from 28 SARS-CoV-2 sequences obtained form 26 patients, together with background SARS-CoV-2 sequences extracted from gisaid that represent viral diversity across the WHO Switzerland region Zurich during the same time frame. Wuhan/WH04/2020 (EPI_ISL_406801), belonging to clade 19B, was chosen as the outgroup. The SARS-CoV-2 lineages are shown in the figure with the patient's sequences labeled with numbers. The scale bar indicates the number of nucleotide substitutions per site.

Fig. S2: Longitudinal assessment of primary and secondary outcomes. (A) Longitudinal clinical assessment of efficacy measures of all trial participants (N=30). Respiratory rate (breaths per minute (bpm)), level of oxygen saturation (SpO2%) and body temperature (°C). (B) Longitudinal laboratory measurements as defined in primary outcomes of all trial participants (N=30): C-reactive protein (CRP), D-dimer, Ferritin, Fibrinogen, IL-6 and LDH. Levels of significance are assessed by paired t-test comparing baseline to subsequent measures. Only significant p-values are shown. Numbers of trial participants with available measurements are indicated.

Fig. S3: No impact of neutralization activity on hospital release. Kaplan-Meier analysis assessing the time to hospital release according to the neutralization level of the plasma received. High neutralization activity NT50>250 (purple), low neutralization activity NT50< 250 (light green). The log-rank test is used to calculate the p-value.

Fig. S4: **Sensitivity analysis for impact of plasma on viral clearance**. (A) Kaplan Meier analysis and (B) survival function estimated with parametric survival model for interval-censored data assessing the time to viral clearance according to neutralization level excluding three trial participants incapable of mounting antibody response (N=27). (C) and (D) corresponding analyses to (A) and (B) but restricting the population to trial participants who did not receive remdesivir (N=26). (E) and (F) corresponding analyses to (A) and (B) but restricting the population to exclude trial participants who received remdesivir or were not capable of mounting antibody response (N=24). (G) and (H) corresponding analyses to (A) and (B) but restricting the population to trial participants who did not receive dexamethasone (N=25). (I) and (J) corresponding analyses to (A) and (B) but restricting the population to exclude trial

participants who received remdesivir or dexamethasone, or were not capable of mounting antibody response (N=20).

Fig. S5: Spike specific binding and neutralizing antibodies are linked with rapid virus clearance. Kaplan-Meier analyses of the effect of the different antibody reactivities stratified by response levels for all combinations of antibody subtypes (IgG, IgA, IgA, IgM) and SARS-CoV-2 antigens (RBD, S1, S2 and NP) as well as the indicated total (summed) reactivities on the time to viral clearance. The effects of neutralization and binding activity as assessed with Roche S on the time to viral clearance are shown in the last row.

Fig. S6: Sensitivity of time to viral clearance when excluding patients on remdesivir. (A) Impact of antibody parameters on the time to viral clearance in multivariable parametric survival models when restricting to trial participants who did not receive remdesivir (N=26). Antibody reactivity of different antibody classes with SARS-CoV-2 antigens and neutralizing antibodies were included. Hazard ratios for individual antibody reactivities adjusted for the presence of comorbidity and the baseline viral load are shown. Significant results are marked in red. Low and high binding activity for each binding antibody parameter is stratified by the respective median binding reactivity. Low and high neutralization activity is stratified by a 50% neutralization titer of 250. (B) Forest plot showing the hazard ratios of the univariable (black) and multivariable (red) model of time to viral clearance in NPS for convalescent donor plasma neutralization level (low and high neutralization activity is stratified by a 50% neutralization titer of 250). Multivariable analyses are corrected for baseline viral load (NPS) and the presence of comorbidity.

Fig. S7:Sensitivity of time to viral clearance when excluding patients on dexamethasone treatment (A) Impact of antibody parameters on the time to viral clearance in multivariable parametric survival models when restricting to trial participants who did not receive dexamethasone (N=25). Antibody reactivity of different antibody classes with SARS-CoV-2 antigens and neutralizing antibodies were included. Hazard ratios for individual antibody reactivities adjusted for the presence of comorbidity and the baseline viral load are shown. Significant results are marked in red. Low and high binding activity for each binding antibody parameter is stratified by the respective median binding reactivity. Low and high neutralization activity is stratified by a 50% neutralization titer of 250.(B) Forest plot showing the hazard ratios of the univariable (black) and multivariable (red) model of time to viral clearance in NPS for

convalescent donor plasma neutralization level (low and high neutralization activity is stratified by a 50% neutralization titer of 250). Multivariable analyses are corrected for baseline viral load (NPS) and the presence of comorbidity.

Fig. S8: Sensitivity of time to viral clearance when excluding patients on dexamethasone, on remdesivir treatment, or incapable of mounting an antibody response. (A) Impact of antibody parameters on the time to viral clearance in multivariable parametric survival models when restricting to trial participants that received neither dexamethasone nor remdesivir and were capable of mounting an antibody response (N=20). Antibody reactivity of different antibody classes with SARS-CoV-2 antigens and neutralizing antibodies were included. Hazard ratios for individual antibody reactivities adjusted for the presence of comorbidity and the baseline viral load are shown. Significant results are marked in red. Low and high binding activity for each binding antibody parameter is stratified by the respective median binding reactivity. Low and high neutralization activity is stratified by a 50% neutralization titer of 250. (B) Forest plot showing the hazard ratios of the univariable (black) and multivariable (red) model of time to viral clearance in NPS for convalescent donor plasma neutralization level (low and high neutralization activity is stratified by a 50% neutralization titer of 250). Multivariable analyses are corrected for baseline viral load (NPS) and the presence of comorbidity.

Fig. S9: No impact on viral clearance when stratifying based on the Elecsys S assay. Forest plot depicting hazard ratios of univariable (grey) and multivariable (black) models of time to viral clearance including Elecsys S assay (U/ml). Multivariable analyses are corrected for baseline viral load and the presence of comorbidity. Low and high binding activity for each binding antibody parameter is stratified by the median binding reactivity.

Fig. S10: **SARS-CoV-2 antibody reactivities are correlated within antibody classes.** Spearman correlation matrix assessing correlation between binding and neutralizing characteristics of the banked plasma samples. Levels of significance are assessed by asymptotic t approximation of Spearman's rank correlation. Color shading denotes correlation coefficient. Stars depict *$p < 0.05$, ** $p < 0.01$, *** $p < 0.001$.

Fig. S11: High neutralizing plasma leads to faster virus decay in nasopharyngeal swabs. (A and B) Censored regression model estimating decay rate of viral load (log10 viral load) of nasopharyngeal swabs from time to

treatment initiation when restricting to trial participants who did not receive remdesivir (N=26), according to (A) the level of neutralization (low neutralization NT50≤250, light green; high neutralization, NT50>250, purple) or (B) level of binding defined by the ABCORA test total S1 SOC values. Low and high total S1 binding is stratified by the median binding reactivity. Significance was assessed using a two-sided t-test.

Fig. S12: **Evolution of SARS-CoV-2 binding antibodies in trial participants**. Longitudinal binding antibody activity of trial participants (N=29) at baseline (day 0, purple), day 9 (green) and day 72 (yellow) assessed with the multiplex SARS-CoV-2 ABCORA test. Depicted are signal over cutoff (SOC) values of IgG, IgA, IgM against RBD and S1, S2 and N. Box plots depict the interquartile ranges with vertical lines representing the minimum and maximum values.

Fig. S13: **Antibody measurements in the three trial participants incapable of mounting an antibody response**. Longitudinal binding antibody activity for the three trial participants assessed with the multiplex SARS-CoV-2 ABCORA test. Depicted are signal over cutoff (SOC) values of IgG, IgA, IgM against RBD, S1, S2 and N.

Fig. S14: **Sensitivity survival analysis adjusting for the impact of endogenous neutralization on virus clearance.** (A) Forest plot showing the hazard ratios from univariable (black) and multivariable (red) models of time to viral clearance for baseline viral load, the presence of comorbidity and both the exogenous and endogenous neutralization, (A) when considering the full cohort (N=29 with available pre-transfusion endogenous neutralization data), and (B) restricting the population to trial participants who did not receive remdesivir (N=25).

Fig. S15: Sensitivity survival analysis adjusting for the impact of endogenous S1 binding antibodies on virus clearance. (A) Forest plot showing the hazard ratios from univariable (black) and multivariable (red) models of time to viral clearance for baseline viral load, the presence of comorbidity and both the exogenous and endogenous total S1 response (sum of IgG, IgA and IgM responses against S1), (A) when considering the full cohort (N=29 with available pre-transfusion data), and (B) restricting the population to trial participants who did not receive remdesivir (N=25).

Figure S1

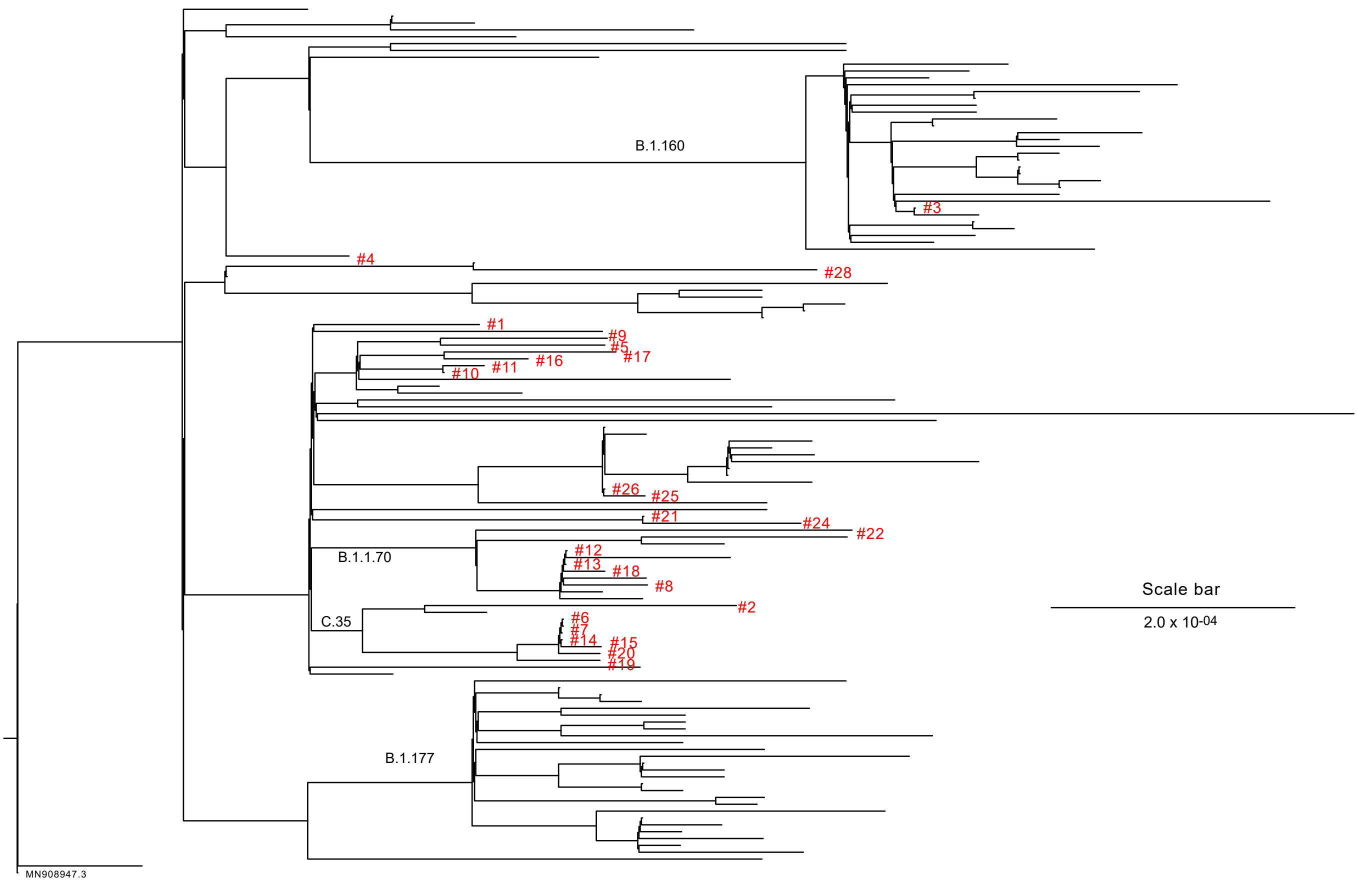
B.1.160
#3
#4
#28
#1
#9
#5
#17
#16
#11
#10
#26
#25
#21
#24
#22
B.1.1.70
#12
#13
#18
#8
#2
C.35
#6
#7
#14
#15
#20
#19
B.1.177
MN908947.3
Scale bar
2.0 x 10-04

Figure S2

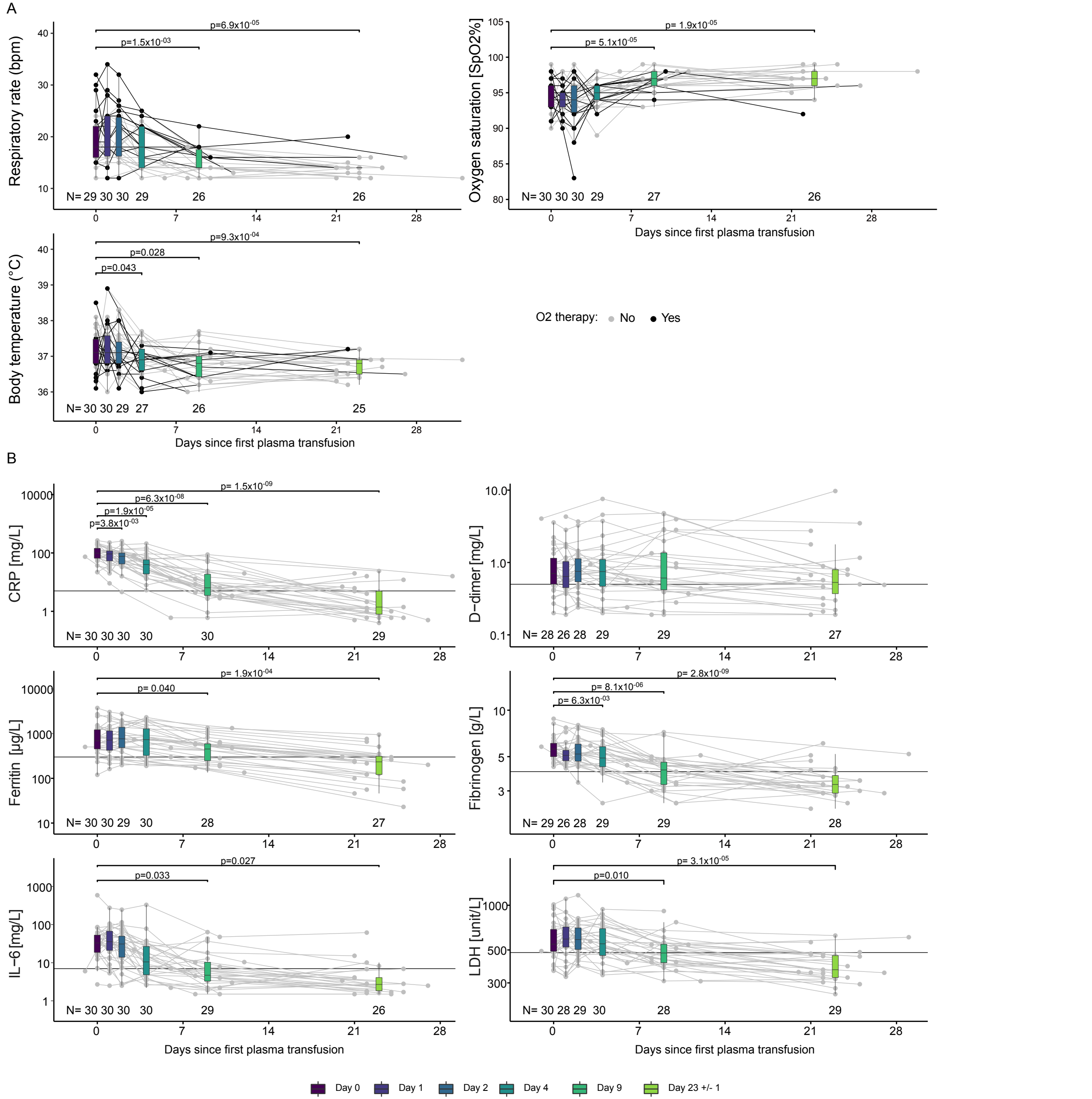
A
Respiratory rate (bpm)
p=6.9x10-05
p=1.5x10-03
N= 29 30 30 29 26 26
Oxygen saturation [SpO2%]
p= 1.9x10-05
p= 5.1x10-05
N= 30 30 30 29 27 26
Days since first plasma transfusion
Body temperature (°C)
p=9.3x10-04
p=0.028
p=0.043
N= 30 30 29 27 26 25
Days since first plasma transfusion
O2 therapy: No Yes
B
CRP [mg/L]
p= 1.5x10-09
p=6.3x10-08
p=1.9x10-05
p=3.8x10-03
N= 30 30 30 30 30 29
D−dimer [mg/L]
N= 28 26 28 29 29 27
Ferritin [µg/L]
p= 1.9x10-04
p= 0.040
N= 30 30 29 30 28 27
Fibrinogen [g/L]
p= 2.8x10-09
p= 8.1x10-06
p= 6.3x10-03
N= 29 26 28 29 29 28
IL−6 [mg/L]
p=0.027
p=0.033
N= 30 30 30 30 29 26
Days since first plasma transfusion
LDH [unit/L]
p= 3.1x10-05
p=0.010
N= 30 28 29 30 28 29
Days since first plasma transfusion
Day 0
Day 1
Day 2
Day 4
Day 9
Day 23 +/- 1

Figure S3

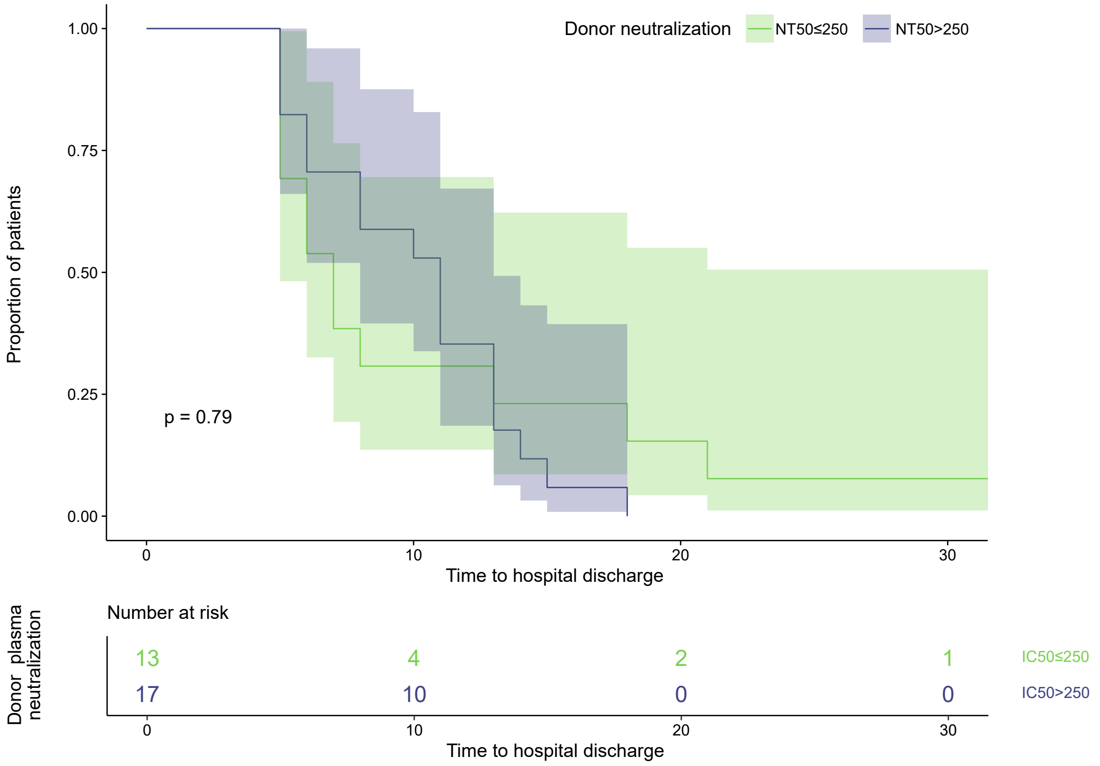

Donor neutralization
NT50≤250
NT50>250
Proportion of patients
1.00
0.75
0.50
0.25
0.00
p = 0.79
0
10
20
30
Time to hospital discharge
Number at risk
Donor plasma neutralization
13
4
2
1
IC50≤250
17
10
0
0
IC50>250
Time to hospital discharge

Figure S4

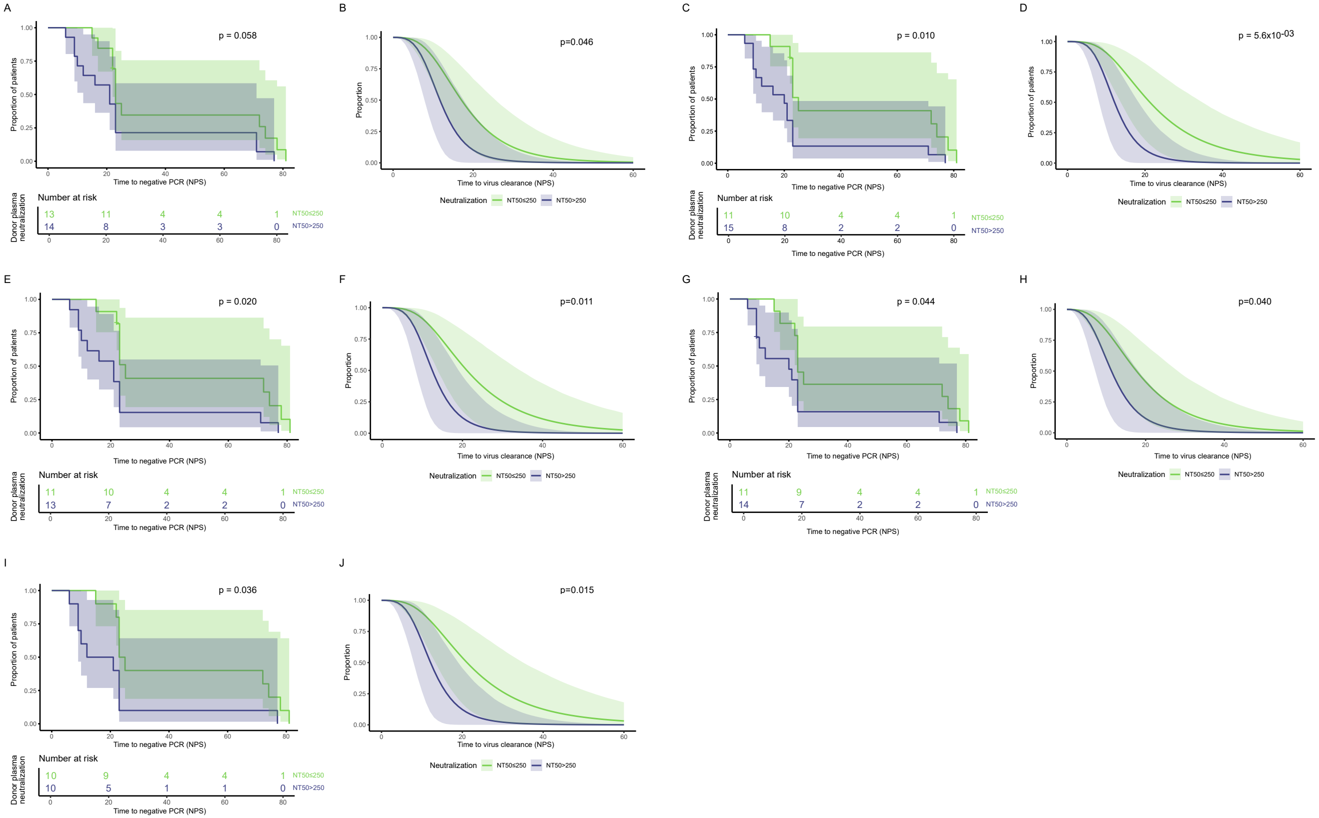

A
p = 0.058
Proportion of patients
Time to negative PCR (NPS)
Number at risk
Donor plasma neutralization
13 11 4 4 1 NT50≤250
14 8 3 3 0 NT50>250
B
p=0.046
Proportion
Time to virus clearance (NPS)
Neutralization NT50≤250 NT50>250
C
p = 0.010
Proportion of patients
Time to negative PCR (NPS)
Number at risk
Donor plasma neutralization
11 10 4 4 1 NT50≤250
15 8 2 2 0 NT50>250
D
p = 5.6x10-03
Proportion of patients
Time to virus clearance (NPS)
Neutralization NT50≤250 NT50>250
E
p = 0.020
Proportion of patients
Time to negative PCR (NPS)
Number at risk
Donor plasma neutralization
11 10 4 4 1 NT50≤250
13 7 2 2 0 NT50>250
F
p=0.011
Proportion
Time to virus clearance (NPS)
Neutralization NT50≤250 NT50>250
G
p = 0.044
Proportion of patients
Time to negative PCR (NPS)
Number at risk
Donor plasma neutralization
11 9 4 4 1 NT50≤250
14 7 2 2 0 NT50>250
H
p=0.040
Proportion
Time to virus clearance (NPS)
Neutralization NT50≤250 NT50>250
I
p = 0.036
Proportion of patients
Time to negative PCR (NPS)
Number at risk
Donor plasma neutralization
10 9 4 4 1 NT50≤250
10 5 1 1 0 NT50>250
J
p=0.015
Proportion
Time to virus clearance (NPS)
Neutralization NT50≤250 NT50>250

Figure S5

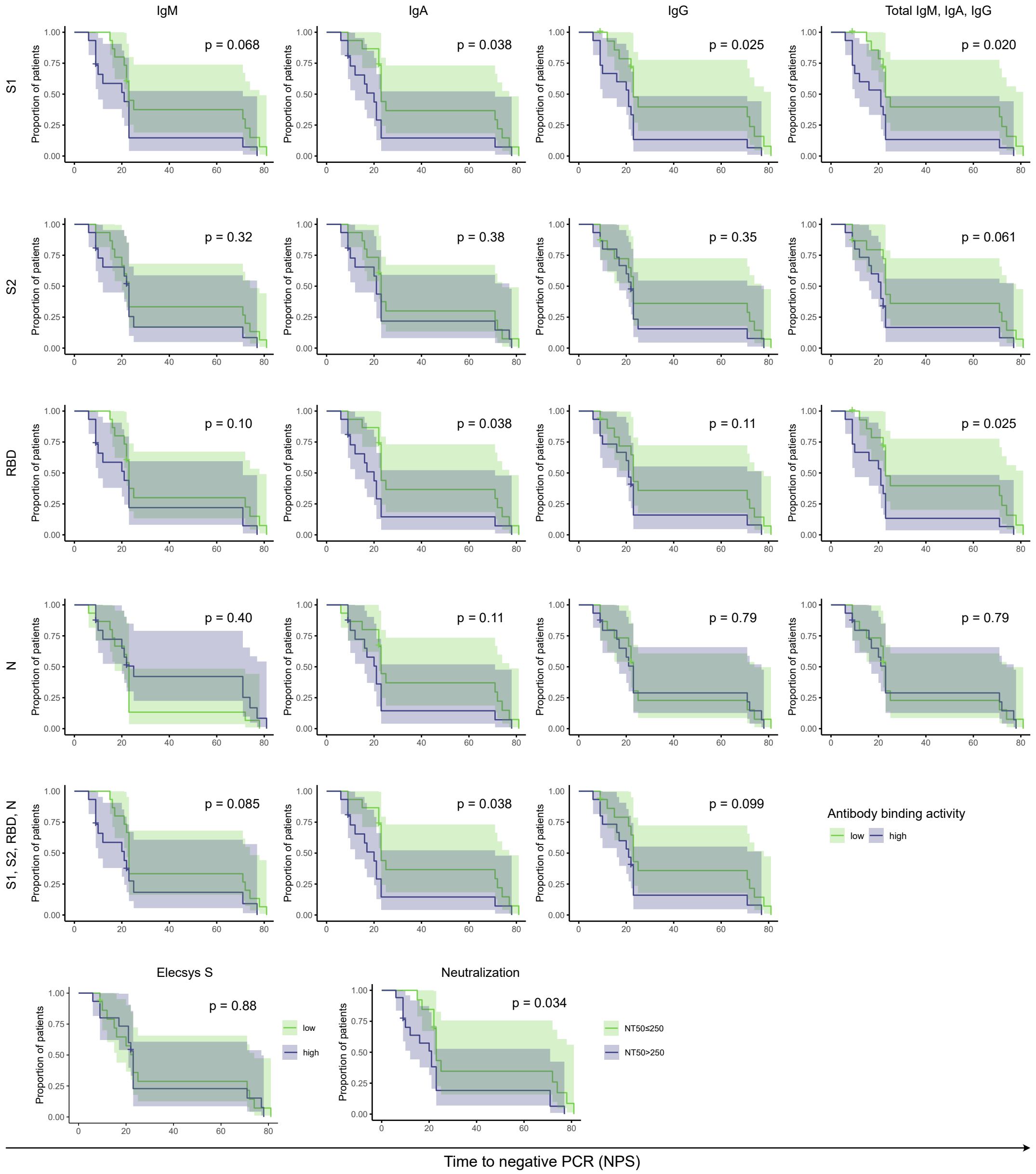
IgM
IgA
IgG
Total IgM, IgA, IgG
S1
S2
RBD
N
S1, S2, RBD, N
Proportion of patients
p = 0.068
p = 0.038
p = 0.025
p = 0.020
p = 0.32
p = 0.38
p = 0.35
p = 0.061
p = 0.10
p = 0.038
p = 0.11
p = 0.025
p = 0.40
p = 0.11
p = 0.79
p = 0.79
p = 0.085
p = 0.038
p = 0.099
Antibody binding activity
low
high
Elecsys S
p = 0.88
low
high
Neutralization
p = 0.034
NT50≤250
NT50>250
Time to negative PCR (NPS)

Figure S6

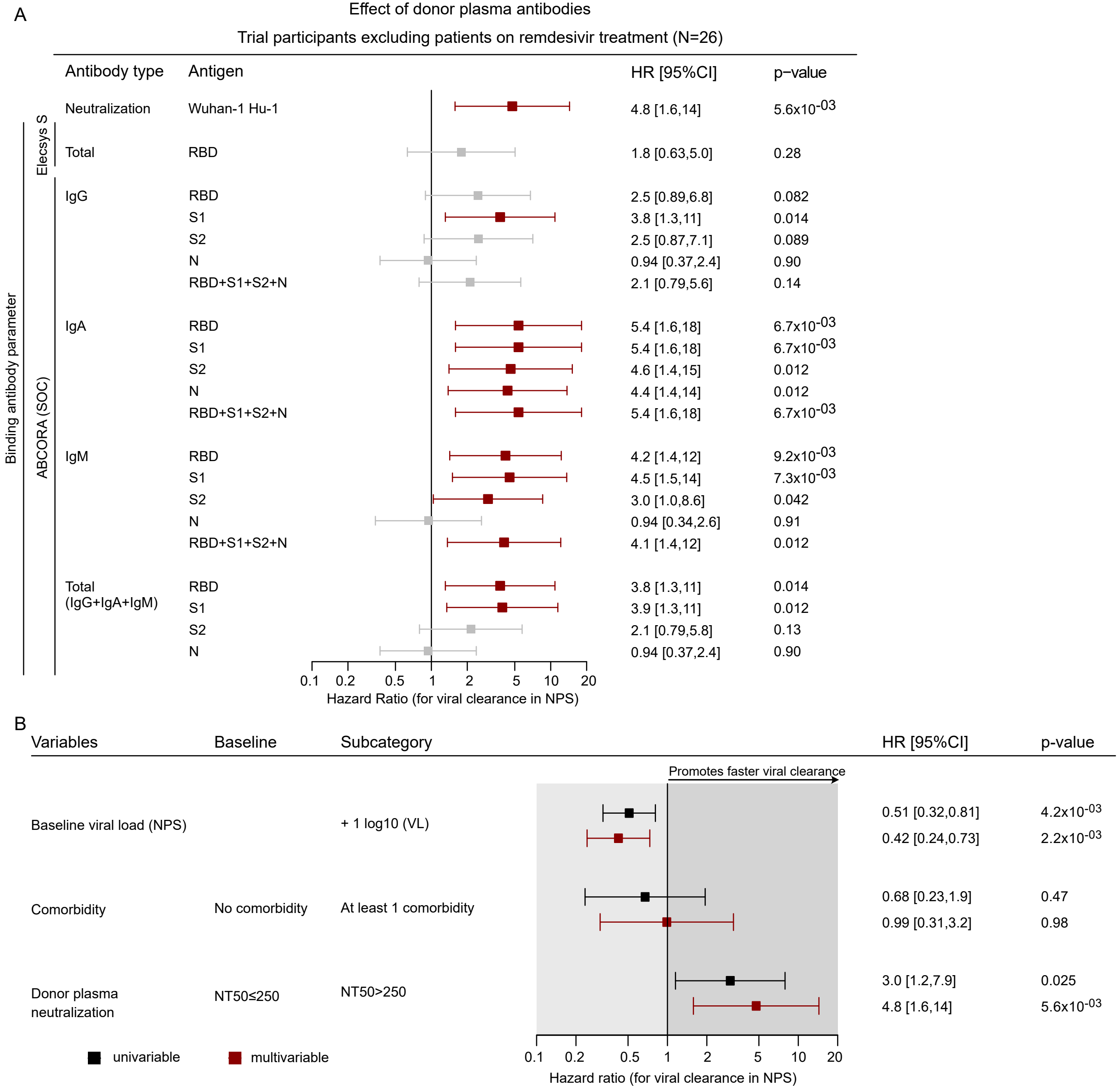

A
Effect of donor plasma antibodies
Trial participants excluding patients on remdesivir treatment (N=26)
Antibody type
Antigen
HR [95%CI]
p−value
Neutralization
Wuhan-1 Hu-1
4.8 [1.6,14]
5.6x10-03
Elecsys S
Total
RBD
1.8 [0.63,5.0]
0.28
Binding antibody parameter
ABCORA (SOC)
IgG
RBD
2.5 [0.89,6.8]
0.082
S1
3.8 [1.3,11]
0.014
S2
2.5 [0.87,7.1]
0.089
N
0.94 [0.37,2.4]
0.90
RBD+S1+S2+N
2.1 [0.79,5.6]
0.14
IgA
RBD
5.4 [1.6,18]
6.7x10-03
S1
5.4 [1.6,18]
6.7x10-03
S2
4.6 [1.4,15]
0.012
N
4.4 [1.4,14]
0.012
RBD+S1+S2+N
5.4 [1.6,18]
6.7x10-03
IgM
RBD
4.2 [1.4,12]
9.2x10-03
S1
4.5 [1.5,14]
7.3x10-03
S2
3.0 [1.0,8.6]
0.042
N
0.94 [0.34,2.6]
0.91
RBD+S1+S2+N
4.1 [1.4,12]
0.012
Total
(IgG+IgA+IgM)
RBD
3.8 [1.3,11]
0.014
S1
3.9 [1.3,11]
0.012
S2
2.1 [0.79,5.8]
0.13
N
0.94 [0.37,2.4]
0.90
0.1 0.2 0.5 1 2 5 10 20
Hazard Ratio (for viral clearance in NPS)
B
Variables
Baseline
Subcategory
HR [95%CI]
p-value
Promotes faster viral clearance
Baseline viral load (NPS)
+ 1 log10 (VL)
0.51 [0.32,0.81]
4.2x10-03
0.42 [0.24,0.73]
2.2x10-03
Comorbidity
No comorbidity
At least 1 comorbidity
0.68 [0.23,1.9]
0.47
0.99 [0.31,3.2]
0.98
Donor plasma
neutralization
NT50≤250
NT50>250
3.0 [1.2,7.9]
0.025
4.8 [1.6,14]
5.6x10-03
univariable
multivariable
0.1 0.2 0.5 1 2 5 10 20
Hazard ratio (for viral clearance in NPS)

Figure S7

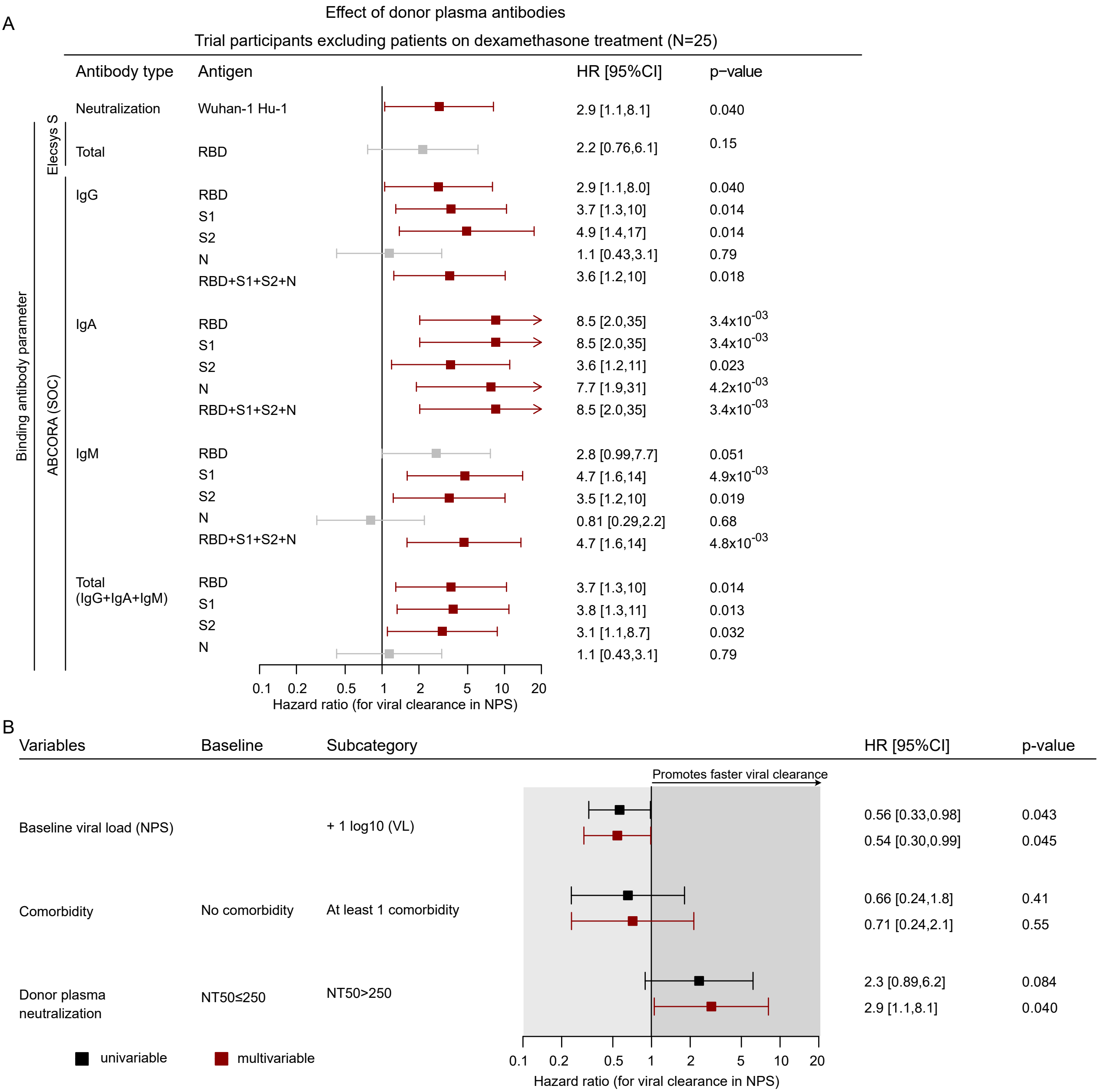
A
Effect of donor plasma antibodies
Trial participants excluding patients on dexamethasone treatment (N=25)
Antibody type
Antigen
HR [95%CI]
p−value
Binding antibody parameter
Elecsys S
ABCORA (SOC)
Neutralization
Wuhan-1 Hu-1
2.9 [1.1,8.1]
0.040
Total
RBD
2.2 [0.76,6.1]
0.15
IgG
RBD
2.9 [1.1,8.0]
0.040
S1
3.7 [1.3,10]
0.014
S2
4.9 [1.4,17]
0.014
N
1.1 [0.43,3.1]
0.79
RBD+S1+S2+N
3.6 [1.2,10]
0.018
IgA
RBD
8.5 [2.0,35]
3.4x10-03
S1
8.5 [2.0,35]
3.4x10-03
S2
3.6 [1.2,11]
0.023
N
7.7 [1.9,31]
4.2x10-03
RBD+S1+S2+N
8.5 [2.0,35]
3.4x10-03
IgM
RBD
2.8 [0.99,7.7]
0.051
S1
4.7 [1.6,14]
4.9x10-03
S2
3.5 [1.2,10]
0.019
N
0.81 [0.29,2.2]
0.68
RBD+S1+S2+N
4.7 [1.6,14]
4.8x10-03
Total
(IgG+IgA+IgM)
RBD
3.7 [1.3,10]
0.014
S1
3.8 [1.3,11]
0.013
S2
3.1 [1.1,8.7]
0.032
N
1.1 [0.43,3.1]
0.79
0.1 0.2 0.5 1 2 5 10 20
Hazard ratio (for viral clearance in NPS)
B
Variables
Baseline
Subcategory
HR [95%CI]
p-value
Promotes faster viral clearance
Baseline viral load (NPS)
+ 1 log10 (VL)
0.56 [0.33,0.98]
0.043
0.54 [0.30,0.99]
0.045
Comorbidity
No comorbidity
At least 1 comorbidity
0.66 [0.24,1.8]
0.41
0.71 [0.24,2.1]
0.55
Donor plasma
neutralization
NT50≤250
NT50>250
2.3 [0.89,6.2]
0.084
2.9 [1.1,8.1]
0.040
univariable
multivariable
0.1 0.2 0.5 1 2 5 10 20
Hazard ratio (for viral clearance in NPS)

Figure S8

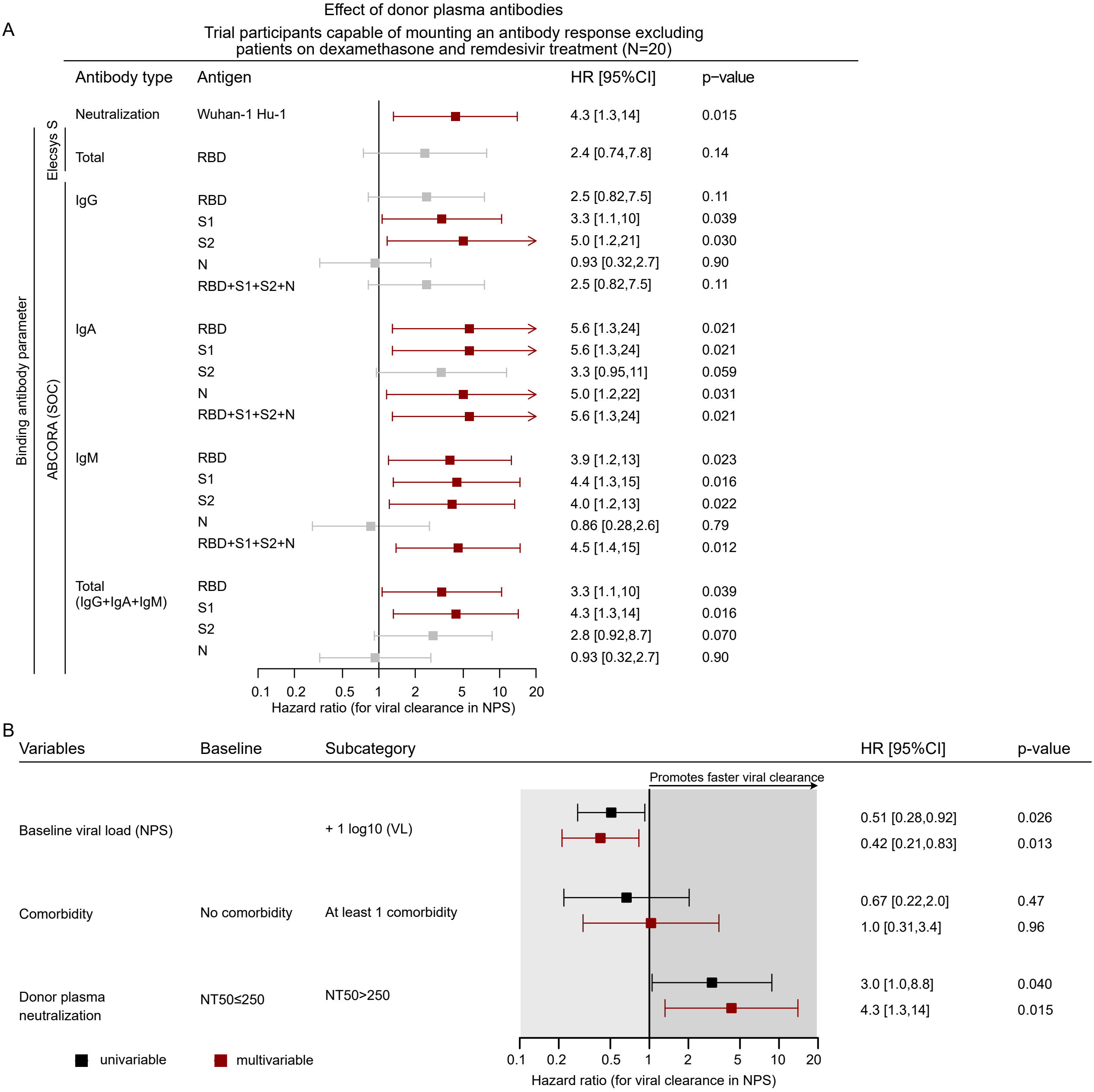

Effect of donor plasma antibodies
A
Trial participants capable of mounting an antibody response excluding patients on dexamethasone and remdesivir treatment (N=20)
Antibody type
Antigen
HR [95%CI]
p−value
Neutralization
Wuhan-1 Hu-1
4.3 [1.3,14]
0.015
Elecsys S
Total
RBD
2.4 [0.74,7.8]
0.14
Binding antibody parameter
ABCORA (SOC)
IgG
RBD
2.5 [0.82,7.5]
0.11
S1
3.3 [1.1,10]
0.039
S2
5.0 [1.2,21]
0.030
N
0.93 [0.32,2.7]
0.90
RBD+S1+S2+N
2.5 [0.82,7.5]
0.11
IgA
RBD
5.6 [1.3,24]
0.021
S1
5.6 [1.3,24]
0.021
S2
3.3 [0.95,11]
0.059
N
5.0 [1.2,22]
0.031
RBD+S1+S2+N
5.6 [1.3,24]
0.021
IgM
RBD
3.9 [1.2,13]
0.023
S1
4.4 [1.3,15]
0.016
S2
4.0 [1.2,13]
0.022
N
0.86 [0.28,2.6]
0.79
RBD+S1+S2+N
4.5 [1.4,15]
0.012
Total (IgG+IgA+IgM)
RBD
3.3 [1.1,10]
0.039
S1
4.3 [1.3,14]
0.016
S2
2.8 [0.92,8.7]
0.070
N
0.93 [0.32,2.7]
0.90
0.1 0.2 0.5 1 2 5 10 20
Hazard ratio (for viral clearance in NPS)
B
Variables
Baseline
Subcategory
HR [95%CI]
p-value
Promotes faster viral clearance
Baseline viral load (NPS)
+ 1 log10 (VL)
0.51 [0.28,0.92]
0.026
0.42 [0.21,0.83]
0.013
Comorbidity
No comorbidity
At least 1 comorbidity
0.67 [0.22,2.0]
0.47
1.0 [0.31,3.4]
0.96
Donor plasma neutralization
NT50≤250
NT50>250
3.0 [1.0,8.8]
0.040
4.3 [1.3,14]
0.015
univariable
multivariable
0.1 0.2 0.5 1 2 5 10 20
Hazard ratio (for viral clearance in NPS)

Figure S9

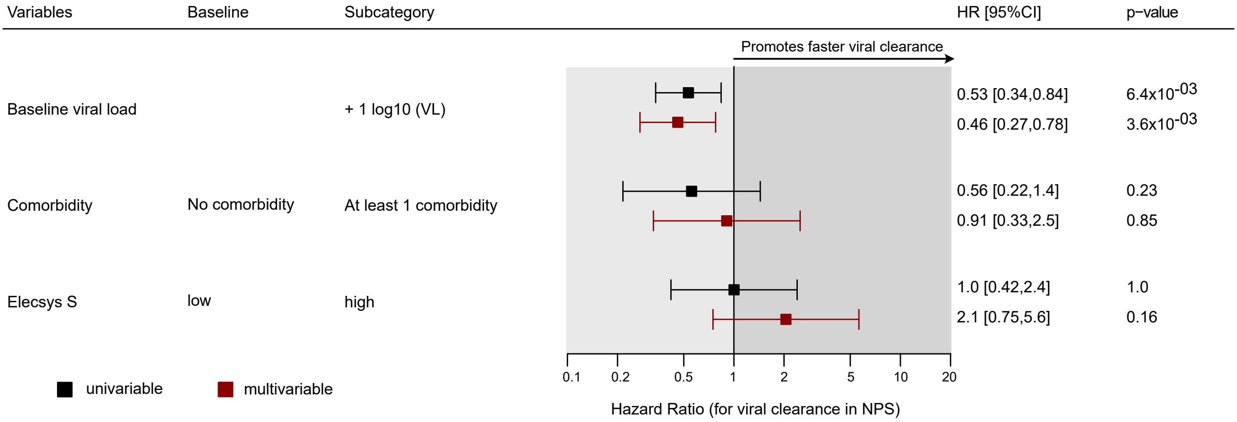
Variables
Baseline
Subcategory
HR [95%CI]
p−value
Promotes faster viral clearance
Baseline viral load
+ 1 log10 (VL)
0.53 [0.34,0.84]
6.4x10-03
0.46 [0.27,0.78]
3.6x10-03
Comorbidity
No comorbidity
At least 1 comorbidity
0.56 [0.22,1.4]
0.23
0.91 [0.33,2.5]
0.85
Elecsys S
low
high
1.0 [0.42,2.4]
1.0
2.1 [0.75,5.6]
0.16
0.1
0.2
0.5
1
2
5
10
20
Hazard Ratio (for viral clearance in NPS)
univariable
multivariable

Figure S10

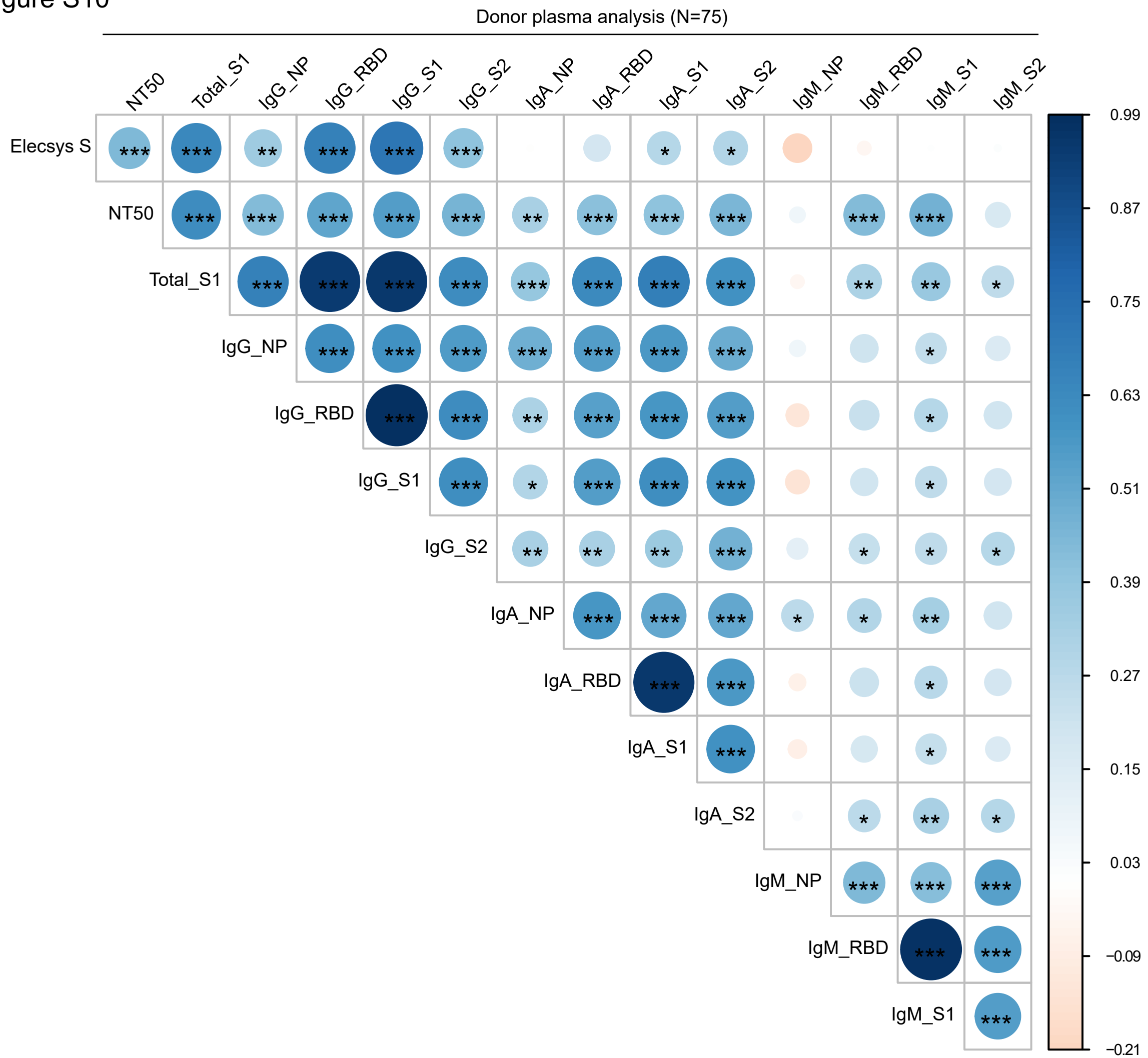
Donor plasma analysis (N=75)
NT50
Total_S1
IgG_NP
IgG_RBD
IgG_S1
IgG_S2
IgA_NP
IgA_RBD
IgA_S1
IgA_S2
IgM_NP
IgM_RBD
IgM_S1
IgM_S2
Elecsys S
0.99
0.87
0.75
0.63
0.51
0.39
0.27
0.15
0.03
−0.09
−0.21

Figure S11

A

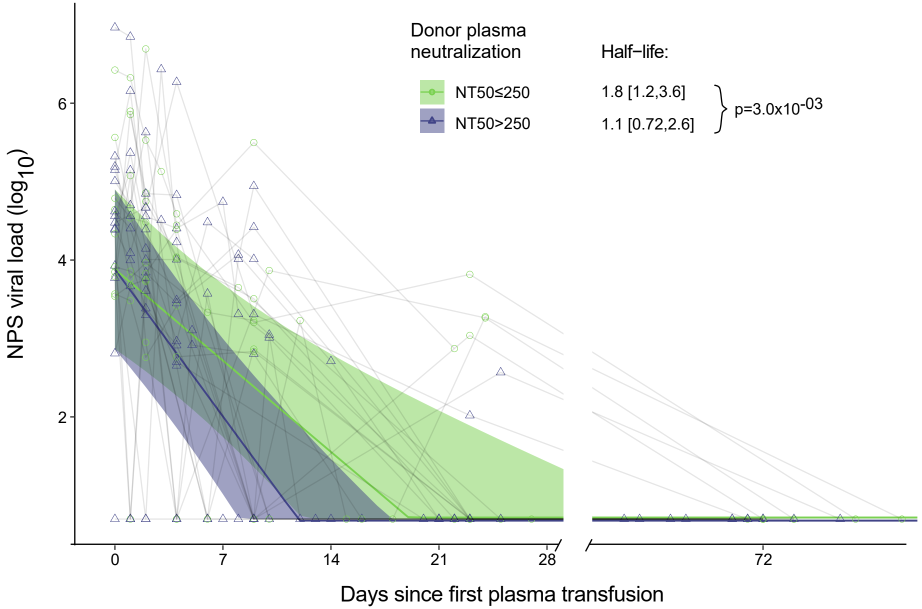

B

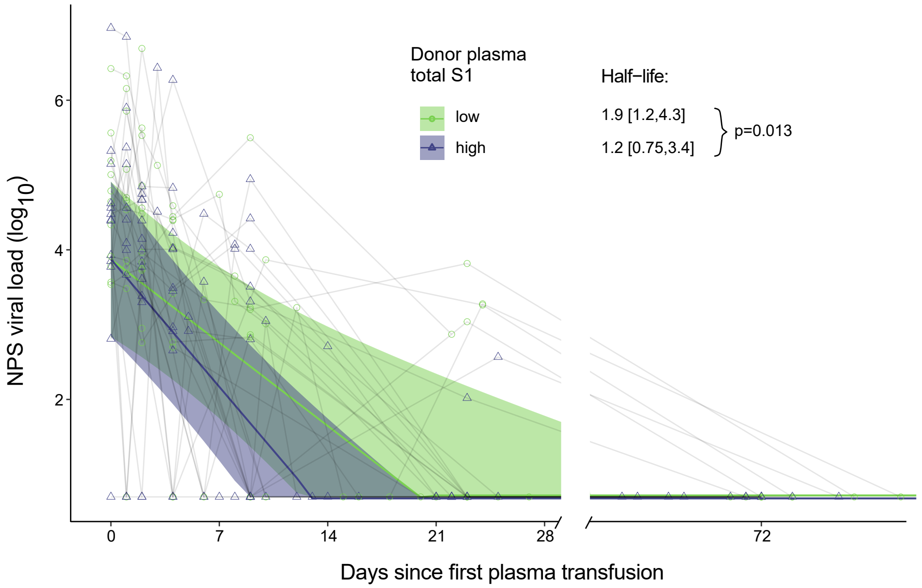

Figure S12

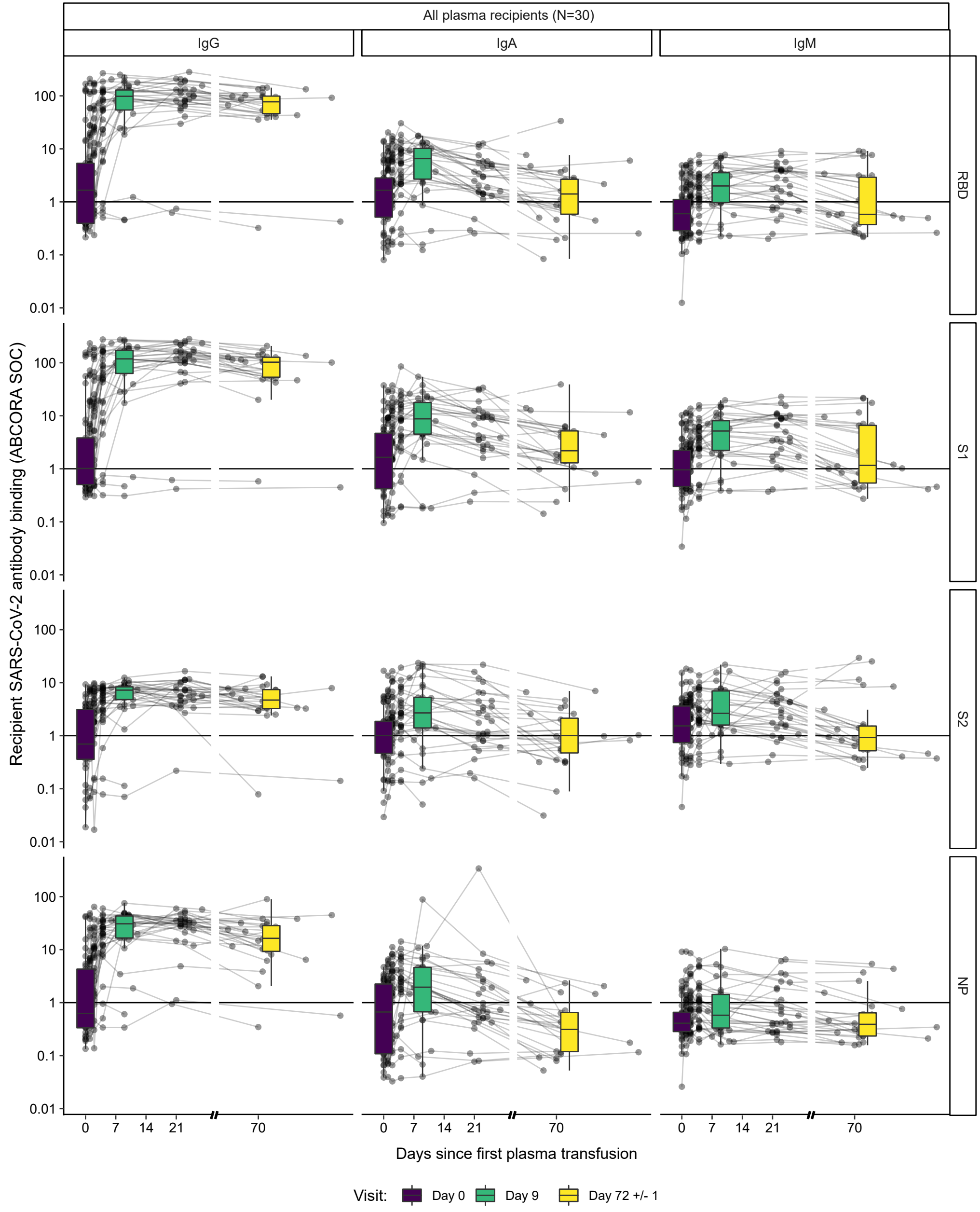

All plasma recipients (N=30)
IgG
IgA
IgM
RBD
S1
S2
NP
Recipient SARS-CoV-2 antibody binding (ABCORA SOC)
100
10
1
0.1
0.01
0
7
14
21
70
Days since first plasma transfusion
Visit:
Day 0
Day 9
Day 72 +/- 1

Figure S13

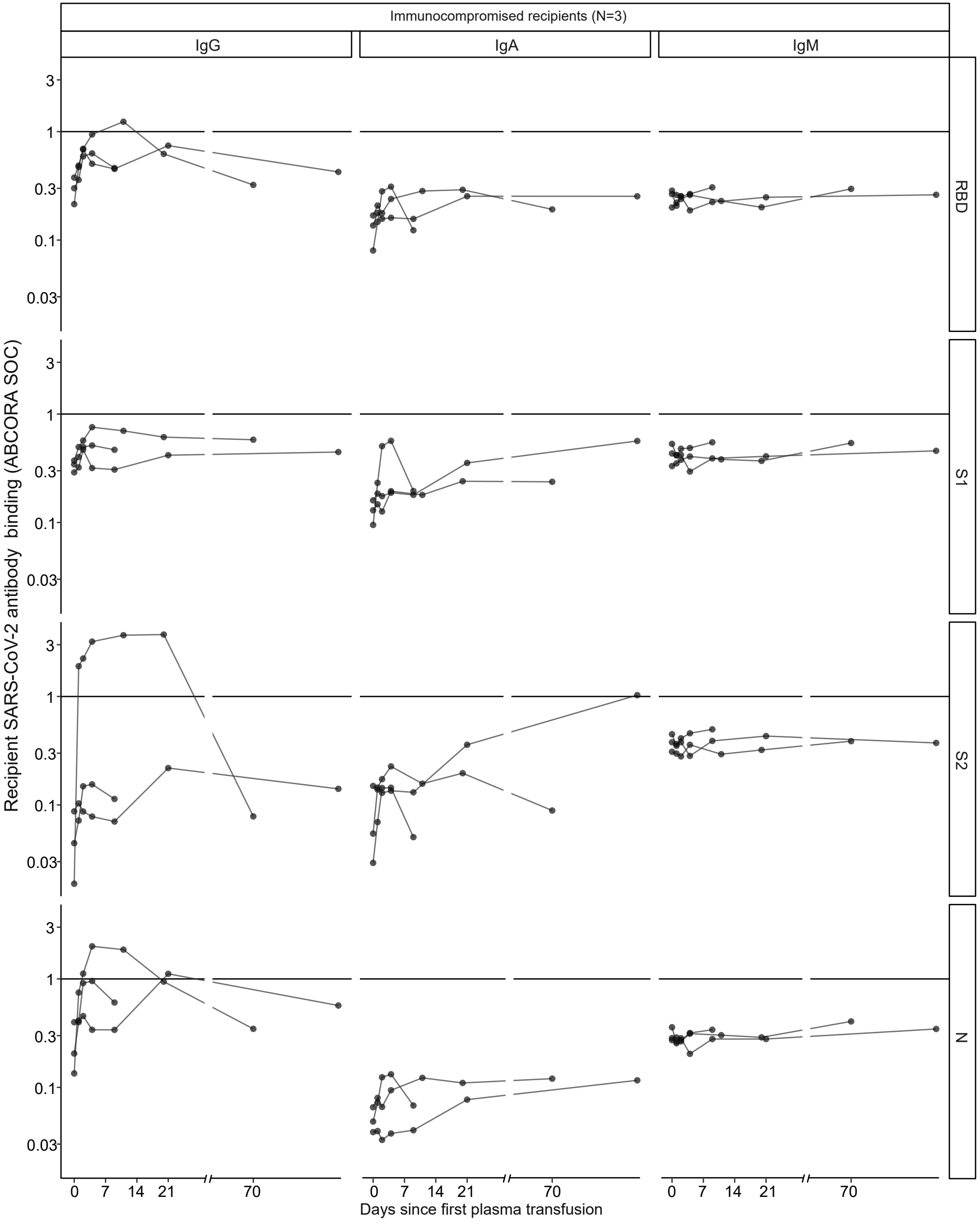
Immunocompromised recipients (N=3)
IgG
IgA
IgM
RBD
S1
S2
N
3
1
0.3
0.1
0.03
Recipient SARS-CoV-2 antibody binding (ABCORA SOC)
0
7
14
21
70
Days since first plasma transfusion

Figure S14

A

All trial participants (N=29)

Variables	Baseline	Subcategory	HR [95%CI]	p-value
Baseline viral load		+ 1 log10 (VL)	0.52 [0.33,0.83]	$6.5x10^{-03}$
			0.49 [0.29,0.83]	$7.4x10^{-03}$
Comorbidity	No comorbidity	At least 1 comorbidity	0.51 [0.20,1.3]	0.17
			0.47 [0.15,1.5]	0.21
Recipient plasma neutralization (pre-transfusion)	NT50≤100	NT50>100	0.56 [0.23,1.4]	0.22
			0.27 [0.080,0.91]	0.035
Donor plasma neutralization	NT50≤250	NT50>250	2.0 [0.80,4.9]	0.14
			4.0 [1.3,13]	0.017

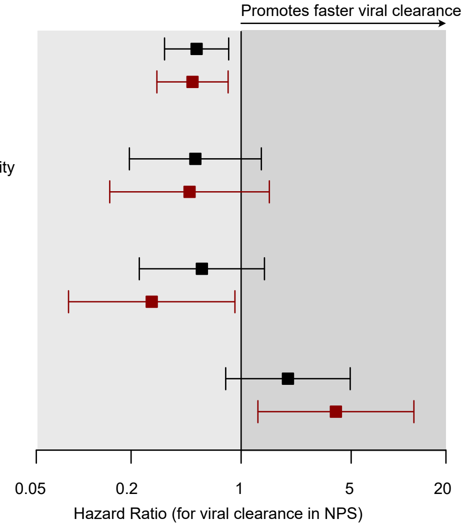

■ univariable ■ multivariable

B

All trial participants without remdesivir treatment (N=25)

Variables	Baseline	Subcategory	HR [95%CI]	p-value
Baseline viral load		+ 1 log10 (VL)	0.50 [0.31,0.80]	$4.1x10^{-03}$
			0.42 [0.23,0.76]	$3.9x10^{-03}$
Comorbidity	No comorbidity	At least 1 comorbidity	0.62 [0.21,1.8]	0.39
			0.77 [0.21,2.8]	0.69
Recipient plasma neutralization (pre-transfusion)	NT50≤100	NT50>100	0.66 [0.25,1.8]	0.41
			0.42 [0.12,1.5]	0.18
Donor plasma neutralization	NT50≤250	NT50>250	2.9 [1.1,7.6]	0.036
			5.2 [1.6,17]	$6.7x10^{-03}$

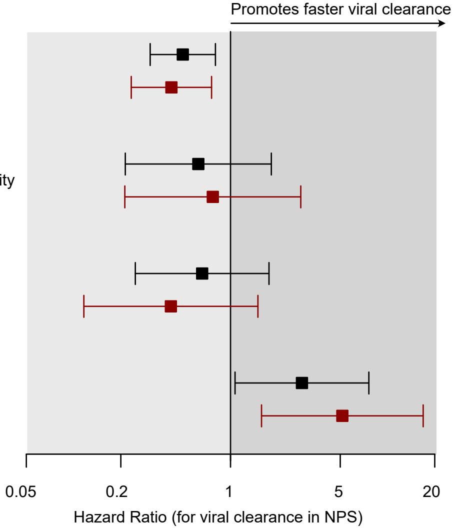

■ univariable ■ multivariable

Figure S15

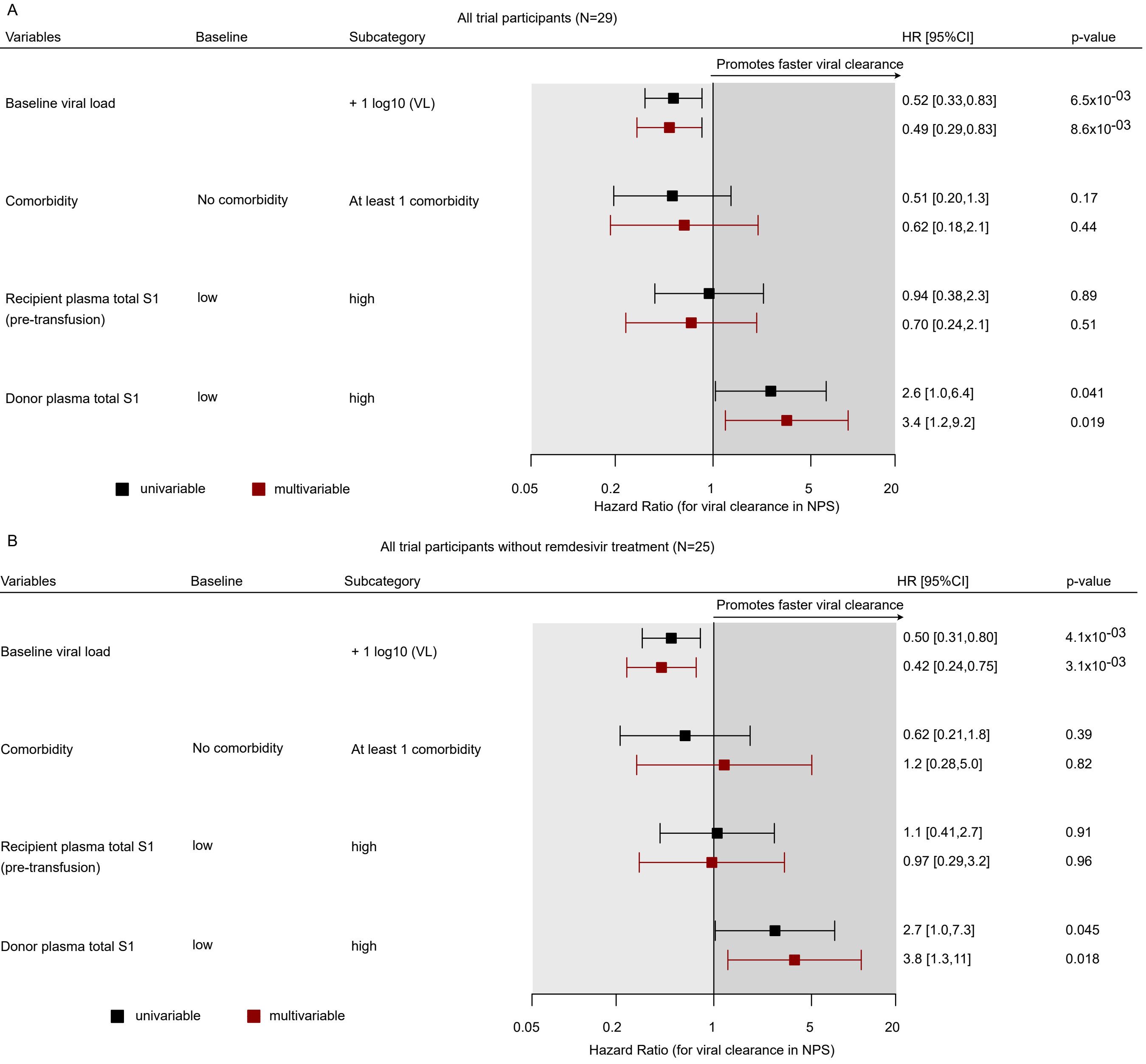
A
All trial participants (N=29)
Variables
Baseline
Subcategory
HR [95%CI]
p-value
Promotes faster viral clearance
Baseline viral load
+ 1 log10 (VL)
0.52 [0.33,0.83]
6.5x10-03
0.49 [0.29,0.83]
8.6x10-03
Comorbidity
No comorbidity
At least 1 comorbidity
0.51 [0.20,1.3]
0.17
0.62 [0.18,2.1]
0.44
Recipient plasma total S1
(pre-transfusion)
low
high
0.94 [0.38,2.3]
0.89
0.70 [0.24,2.1]
0.51
Donor plasma total S1
low
high
2.6 [1.0,6.4]
0.041
3.4 [1.2,9.2]
0.019
univariable
multivariable
0.05
0.2
1
5
20
Hazard Ratio (for viral clearance in NPS)
B
All trial participants without remdesivir treatment (N=25)
Variables
Baseline
Subcategory
HR [95%CI]
p-value
Promotes faster viral clearance
Baseline viral load
+ 1 log10 (VL)
0.50 [0.31,0.80]
4.1x10-03
0.42 [0.24,0.75]
3.1x10-03
Comorbidity
No comorbidity
At least 1 comorbidity
0.62 [0.21,1.8]
0.39
1.2 [0.28,5.0]
0.82
Recipient plasma total S1
(pre-transfusion)
low
high
1.1 [0.41,2.7]
0.91
0.97 [0.29,3.2]
0.96
Donor plasma total S1
low
high
2.7 [1.0,7.3]
0.045
3.8 [1.3,11]
0.018
univariable
multivariable
0.05
0.2
1
5
20
Hazard Ratio (for viral clearance in NPS)
